# Sustainable Self-Healing Thermoresponsive Nanocomposite Derived from Shrimp Shells and Lignin Biowaste for Water Shutoff in Ultradeep High-Temperature, High-Salinity Carbonate Reservoirs

**DOI:** 10.1021/acsomega.6c08760

**Published:** 2026-10-28

**Authors:** Shahenda Mahran, Basudeb Saha

**Affiliations:** † Faculty of Energy and Environmental Engineering, 120633 The British University in Egypt, Misr-Ismailia Road, El-Sherouk City, Cairo 11837, Egypt; ‡ School of Engineering, 4396 Lancaster University, Lancaster LA1 4YR, U.K.

## Abstract

Excessive water production in ultradeep, extreme high-temperature and high-salinity (HTHS) carbonate reservoirs has emerged as a major challenge, reducing hydrocarbon productivity and creating substantial environmental and economic burdens. Herein, we report, for the first time, the synthesis of a novel, highly resilient, sustainable, self-healing, thermoresponsive chitin-based nanocomposite hydrogel for water shutoff, specifically tailored to endure extreme harsh conditions of 160 °C and 350,000 ppm salinity. The nanocomposite was synthesized via a near-zero-waste approach utilizing chitin and CaCO_3_ nanoparticles derived from shrimp shell biomass, employing a recyclable, CO_2_-switchable natural deep eutectic solvent (NADES)-assisted enzymatic process. The extracted chitin was copolymerized with vinyl ester sulfonated lignin (VESL), a green macromonomer derived from industrial lignin waste, and 1-vinylimidazole (VIm) via free radical polymerization. The hydrogel composition has been optimized using response surface methodology (RSM) with central composite design. The nanocomposite was thoroughly characterized by FTIR, ^1^H NMR, SEM, EDX, TGA, and DTG analysis. The optimized nanocomposite demonstrated a superior swelling ratio and viscoelastic properties even at an ultralow polymer concentration of 0.08 wt % across temperatures of 25 to 160 °C and salinities up to 350,000 ppm. Core-flooding experiments indicated effective fracture plugging with high RRF values of 300.5 ± 5.7 and 191.1 ± 4.8 (with plugging efficiencies of 99.7 ± 0.4% and 99.5 ± 0.5%, respectively) using ultralow nanocomposite concentrations of 0.08 wt % and 0.1 wt %, respectively. This research presents a sustainable strategy that integrates the CO_2_-switchable green extraction of chitin with lignin for valorization of marine and industrial biowaste into a high-performance self-healing hydrogel for water shutoff in ultra-HTHS reservoirs.

## Graphical abstract

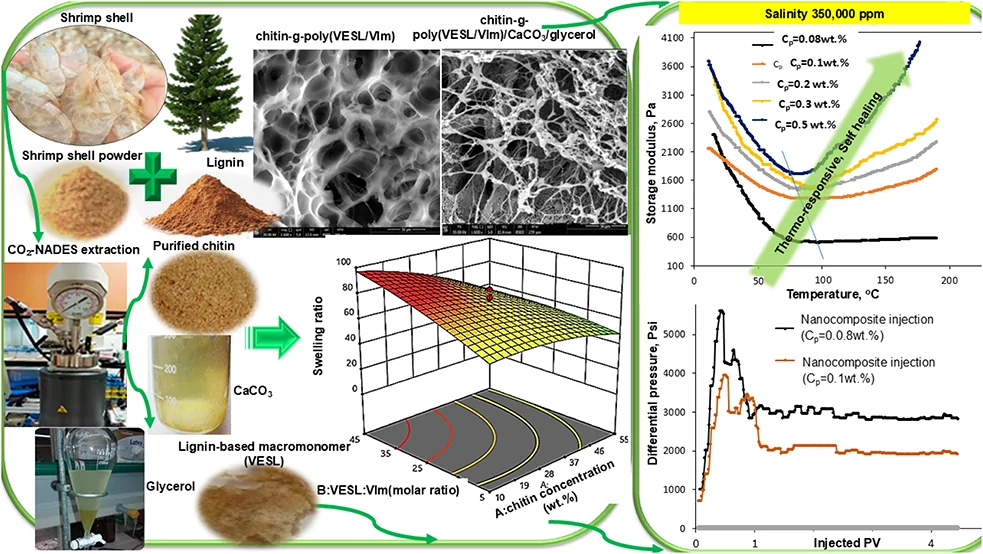

## 1 Introduction

With the quick progress in materials science and the increasing focus on environmental sustainability, there is significant attention on the valorization of green feedstock for the synthesis of robust and green materials, which attracts both academic and industrial attention.^1–4^ In the long run, different biomass materials have been utilized as sustainable resources to produce value-added polymeric materials with adaptable mechanical properties.^5–8^ Although the utilization of cellulose,^9–12^ lignin,^13–16^ and lipids^17,18^ has been intensively evaluated, the chemical conversion of chitin into value-added polymers is still at a nascent stage, particularly via green and zero-waste routes. Furthermore, even though lignin is one of the most abundant biopolymers, its structural heterogeneity limits its wide applications. Lignin is an aromatic, sustainable source that is extracted from various wood types and utilized as an organic filler to synthesize polymeric materials with improved performance.^16,19,20^ Furthermore, it can be converted to different aromatic chemicals through depolymerization techniques into value-added materials.^21,22^ Recently, lignin-based monomers such as methacrylate and acrylate have been widely evaluated for the synthesis of eco-friendly polymeric networks for advanced adhesives, coatings, and resins.^23–25^ Owing to the aromatic groups, lignin-derived polymers demonstrate excellent mechanical properties, which can further extend their applications.^26,27^ Chitin exists in crustacean shells, i.e., crab and shrimp shells, considered the second most abundant biomass resource after cellulose, with a global production of about 100 billion tons.^28–30^ However, extraction of chitin from shellfish wastes using green, zero-waste approaches, particularly via CO_2_-switchable NADES-assisted enzymatic extraction, remains unreported. The extraction of chitin from shellfish wastes and its conversion into valuable chemicals instead of its disposal in landfills and oceans offer both environmental and economic benefits.^31^ Chitin and its deacetylated form of chitosan are interesting alternatives to synthetic polymers owing to their nontoxicity, low cost, and availability.^32,33^ The presence of hydroxyl groups, carboxylic acids, and amines is utilized in cross-linking or grafting other polymers.^34^ Nevertheless, their applications are challenging owing to their poor stability and low mechanical strength, particularly at high temperatures.^35,36^ The literature has reported many attempts at grafting chitin with synthetic polymers, intending to improve their physicochemical properties.^37^ Grafted chitin has been widely utilized as a superabsorbent or hydrogel owing to its capability to create hydrogen bonds and to be ionized in water, which forms a pressure gradient involving water diffusion into its structures and swelling.^38,39^ The integration of chitin and lignin offers a promising approach to overcoming individual limitations and formulating a novel green hybrid hydrogel nanocomposite with improved chain entanglements and mechanical properties. Although chitin- and lignin-based hydrogels have been widely investigated in the literature for several fields, i.e., biomedical applications, water treatment, seed coating, and adsorbents for industrial waste,^39–44^ their potential for the synthesis of a self-healing, thermoresponsive hydrogel via CO_2_-switchable NADES-extracted chitin for water shutoff application in ultraharsh carbonate oil reservoirs has not been reported.

Excess water production has been a significant issue in the oil industry, leaving behind a substantial amount of unproduced hydrocarbon in a reservoir. This excess water reduces the lifetime of oil wells,^45^ accelerates corrosion scale deposition, and greatly strains surface facilities.^46^ Recent statistics indicate that 210 million barrels of water are produced daily worldwide, per 75 million barrels of oil produced.^47^ Many of the oilfields with water flooding in the mid-to-late stages are characterized by severe water channels formed owing to reservoir heterogeneity and long-term water flooding, resulting in a significant reduction in the sweep efficiency of the injected fluids.^48^ This water typically originates from active aquifers or is produced alongside oil during the final stage of water flooding.^49–51^ Growing attention has been given to oil reservoirs with open-hole fractures where the production well is linked to an aquifer by a conductive fracture, resulting in a significant reduction in oil recovery, where the injected fluids bypass oil zones, a phenomenon known as thief zones.^52^ This challenge is critical in ultradeep marine carbonate reservoirs, such as the Shunbei and Tahe fields in the Tarim Basin, as well as significant fields in the Middle East. At depths exceeding 7,000 m, operations face extreme conditions with temperatures reaching up to 160 °C and salinities exceeding 350,000 ppm. Due to the rapid thermal breakdown and chemical instability of standard polymer gels in these ultrahigh-temperature, high-salinity (HTHS) environments, there is an immediate need in the industry for robust, sustainable plugging materials.^53^

The literature has reported different attempts at the application of bulk gel systems, elastic polymers, and foams for water production control in sandstone reservoirs, which demonstrated promising results in profile control of these reservoirs.^45,54–56^ Different authors reported the synthesis of various gel systems through the utilization of polyacrylamide, poly­(acrylic acid), and metallic or organic cross-linkers such as N,N′-methylene-bis­(acrylamide)^57–62^ and sulfonated polyacrylamide with inorganic cross-linkers, including aluminum nitrate nonahydrate,^63^ chromium acetate,^64^ and polyethylenimine^65^ to form preformed particle gels (PPG), polymer microspheres,^66,67^ microgels,^68,69^ and dispersed particle gels (DPG).^70^ These hydrogels were synthesized through the polymerization of anionic, cationic, and amphoteric acrylamide-based monomers and cross-linkers.^71,72^ Other researchers reported the synthesis of PPGs through the polymerization of anionic sulfonated polyacrylamide with chromium acetate, with a high gel strength of about 35.4 kPa; however, results indicated their limited stability at 80 °C and a TDS of 149,829 ppm. Furthermore, the usage of chromium acetate as a highly hazardous and toxic material restricted its field application. Paprouschi et al. and Khoshkar et al.^73,74^ reported the synthesis of thermally stable nano-PPG through the incorporation of graphene oxide into their polymeric chains, where the effectiveness of the nano-PPG in increasing the sweep efficiency of water injection and improving oil production was proven only in low-salinity water flooding up to 80 °C. Zhongjian et al.^75^ introduced a soft microgel with a salinity resistance of only 5000 mg/L. In these studies, the rheological properties of the synthesized hydrogels were evaluated at medium and high reservoir temperatures ranging from 60 to 120 °C and average salinities less than 150,000 mg/L. However, these hydrogels cannot withstand the extremely harsh reservoir conditions of ultradeep fractured carbonate with total dissolved ions higher than 350,000 ppm and the reservoir temperature higher than 150 °C, demonstrating breakage of molecular chains leading to destruction of the polymeric structure and polymer chain degradation, which facilitates gel dehydration and hence poor plugging performance.

Recently, research has been intensified to overcome the limitations of PPG systems to make them more resistant to ultradeep fractured carbonate reservoirs that exist in the Gulf countries, distinguished by extremely harsh conditions of HTHS often higher than 150 °C. Baloochestanzadeh et al.^62^ reported the synthesis of a starch-grafted polyacrylamide cross-linked hydrogel, which was resistant up to a temperature of 90 °C and a TDS of 225,000 ppm. However, these PPGs demonstrated limited stability at temperatures higher than 90 °C owing to inadequate strength to attain long-term thermal stability. Other researchers evaluated the plugging performance of different polymeric systems, such as polymer gels using metal cross-linkers, organic gel systems, PPG, and DPG, which demonstrated moderate plugging performance at high temperatures (140 °C) and in formation water (240,000 mg/L). Consequently, at ultraharsh conditions of temperatures higher than 150 °C, these hydrogels undergo chemical and thermal degradation. In addition, there are raised environmental issues owing to the toxicity of used cross-linkers and other chemicals used in the synthesis of these hydrogels. Therefore, it is crucial to design a novel sustainable hydrogel with high stability at ultradeep fractured carbonate reservoirs.

Recent efforts to improve the thermal stability and mechanical strength of PPGs have been directed toward self-healing and thermoresponsive hydrogels. Self-healing hydrogels enable the regeneration of distracted particle gels through recross-linkable connections using dynamic cross-links such as hydrophobic interactions, electrostatic forces, and hydrogen bonding. Can et al.^76^ reported that the synthesis of self-healing hydrogels can be achieved through copolymerization of hydrophobic stearyl methacrylate with long alkyl chains, which contribute to hydrophobic associations of long alkyl groups. Furthermore, hydrogels with β-cyclodextrin content have self-healing properties owing to the formation of supramolecular inclusion complexes.^77^ Despite the improved mechanical properties of self-healing hydrogels, they fail to reassemble into a bulk network from microgel particles in saline media. However, amphoteric hydrogels can cross-link into macroscale gels within saltwater by exploiting reversible ionic bridges.^78–80^ Ionically cross-linked gels can be re-cross-linked to form a bulk gel, which makes it appropriate for plugging fractures in oil reservoirs. In contrast to traditional PPGs, which cannot re-cross-link and form bulk gels from particle gels, re-cross-linkable PPGs can produce bulk gels with mechanical strength similar to that of conventional PPGs, through the use of a polyion complex hydrogel approach. In parallel, thermosensitive hydrogels can be categorized according to temperature-sensitive phase transitions into LCST (Lower Critical Solution Temperature)^81^ and UCST (Upper Critical Solution Temperature).^82^ LCST hydrogels demonstrate a transition from hydrophilic to hydrophobic behavior as temperature increases, whereas UCST hydrogels demonstrate the reverse transition. Compared to LCST hydrogels, UCST hydrogels are less investigated and are mostly controlled by hydrogen bonding and Coulombic interactions (e.g., amphoteric polymers), which trigger their temperature responsiveness.^83,84^ Recent studies have demonstrated that these hydrogels can be adopted for improved mechanical properties and multifunctionality.^85,86^ Zhou et al.^87^ have reported that the sol–gel transition temperatures of poly­(acrylamide-*co*-acrylonitrile)-*b*-poly­(poly­(ethylene glycol)­methyl ether methacrylate) can be tuned through modifying chain length and concentration. Furthermore, dual-network hydrogels combining LCST and UCST behaviors have been synthesized, leading to enhanced mechanical strength, toughness, and self-healing properties. Despite these advancements, these synthetic hydrogels are still fundamentally reliant on petroleum-derived acrylamide or vinyl networks cross-linked via toxic organic agents. When subjected to extreme conditions that exceed 150 °C and 300,000 ppm TDS, these synthetic structures commonly experience significant charge shielding, chemical degradation, or drastic volume syneresis. As a result, the synthesis of a sustainable thermoresponsive self-healing hydrogel that does not compromise matrix injectivity, thermal durability, or long-term plugging effectiveness under extreme downhole conditions remains a challenge that has yet to be addressed. To clearly illustrate these shortcomings, a detailed comparison of existing water-shutoff hydrogels in terms of their operational limits and compositions will be provided in the later part of the manuscript. According to the literature, no previous studies have been reported so far for the synthesis of a green, self-healing, thermoresponsive hydrogel using a near-zero-waste, cost-effective route for water shutoff application of ultradeep fractured carbonate reservoirs at extremely harsh reservoir conditions of total dissolved ions of 350,000 ppm and reservoir temperatures up to 160 °C.

To address the identified limitations, this research reports for the first time the synthesis of a novel sustainable, chitin-based, self-healing, thermoresponsive nanocomposite hydrogel for water shutoff applications in ultradeep fractured carbonate reservoirs under ultraharsh carbonate reservoir conditions (160 °C, 350,000 ppm TDS) via near-zero-waste valorization of shrimp shells and lignin waste. Chitin and CaCO_3_ nanoparticles were extracted from shrimp shells via a zero-waste CO_2_-switchable, NADES-assessed enzymatic extraction and utilized with glycerol as a green cross-linker. The adaptable characteristics of NADES facilitate effective solvent recovery and reuse, greatly improving the economic feasibility and industrial scalability of the extraction method. The extracted chitin was then grafted with a green sulfonated lignin macromonomer and VIm, utilizing upcycled biodiesel-derived glycerol as a green cross-linker and the extracted CaCO_3_ nanoparticles as green functional reinforcements. The composition of the synthesized hydrogel was systematically optimized using response surface methodology (RSM) and central composite design (CCD), investigating the effect of chitin content, VESL:VIm ratio, CaCO_3_ loading, and glycerol concentration on swelling ratio and viscoelastic properties. Further, the synthesized hydrogel has been extensively characterized by FTIR, ^1^H NMR, TGA-DTG, TEM, SEM, and DLS techniques. The synthesized hydrogel was evaluated for water shutoff application by conducting displacement experiments using carbonate cores at simulated hostile reservoir conditions of 160 °C and a salinity of about 350,000 ppm, and the RRF and the plugging efficiency were determined. To the best of our knowledge, this is the first research on a green self-healing, thermoresponsive hydrogel derived from shrimp shells and agricultural waste streams using a zero-waste CO_2_-switchable NADES-assisted enzymatic extraction approach for water shutoff applications in ultradeep fractured carbonate reservoirs under ultraharsh carbonate reservoir conditions (160 °C, 350,000 ppm TDS). This research offers a highly robust, sustainable, self-healing, and thermoresponsive nanocomposite hydrogel for water shutoff applications under ultraharsh carbonate reservoir conditions, while introducing an innovative valorization pathway for seafood, agricultural wastes, and biodiesel byproducts into value-added polymeric materials. Through the conversion of marine and agricultural industrial waste into high-performance polymeric materials, this research provides a strong and economically viable strategy for application-driven biomass valorization.

## 2 Experimental Section

### 2.1 Materials

Lignin; shrimp shells; triethylamine (TEA, 99.5%, Aladdin); vinylsulfonyl chloride; N,N-dimethyl sulfoxide (DMSO ≥ 99.9%); anhydrous sodium carbonate (Na_2_CO_3_ ≥ 99.99%); anhydrous sodium sulfate (Na_2_SO_4_); anhydrous dichloromethane (DCM ≥ 99.8%); ethyl acetate (≥99%); alcalase; bromelain (≥3 units/mg protein); 4-(dimethylamino)­pyridine (DMAP ≥ 98.0%); 1-vinylimidazole (VIm ≥ 99%); potassium persulfate (KPS ≥ 99%); dimethyl sulfoxide-d_6_ (99.9 atom %D); ethanol ultrapure; acetone (≥97%); choline chloride (**≥**99%); 2-(diethylamino)­ethanol (**≥**99.5%); and recycled glycerol were used. All chemicals were supplied by Sigma-Aldrich.

### 2.2 CO_2_-Switchable NADES-Based Enzymatic Extraction of Chitin and CaCO_3_ Nanoparticles from Shrimp Shells

Shrimp shells were dried at 60 °C for 24 h and ground into fine powder. A natural deep eutectic solvent (NADES) was prepared by mixing choline chloride and glycerol (1:2 molar ratio) at 60 °C for 2 h until a homogeneous liquid was formed. 100 g of dried shrimp shell powder was suspended in 500 mL of diluted NADES liquid (30% v/v), followed by the addition of 2% (w/v) of alcalase and bromelain enzymes for enzymatic deproteinization. The reaction mixture was placed in a thermostatic shaking incubator at 54 °C for 3 h. The solid residue composed of chitin and CaCO_3_ was separated by centrifugation, washed with water, and suspended again in a switchable water system containing 2-(diethylamino)­ethanol. This reaction mixture was moved to a high-pressure reactor with CO_2_ at 4 bar and 25 °C for 3 h for demineralization of CaCO_3_ via in situ formation of carbonic acid. The reactor was then gradually depressurized to ambient pressure, and the reaction mixture was centrifuged; the solid chitin was separated from the protein hydrolyzate and washed with distilled water. After the separation of chitin, the liquid filtrate, carbon dioxide-rich gas, was further subjected to N_2_ purging for 2 h at 40 °C until the pH reached 9. The precipitated CaCO_3_ nanoparticles were filtered, washed with water, and dried at 45 °C.

### 2.3 One-Pot Synthesis of Vinyl Ester Sulfonated Lignin Macromonomer (VESL)

The synthesis of vinyl ester sulfonated lignin was performed using a one-pot, in situ transesterification reaction between ethyl vinyl sulfonate and lignin. For the synthesis of ethyl vinyl sulfonate, a 250 mL double-wall reactor equipped with a nitrogen inlet and outlet was charged with 25 mL of anhydrous dichloromethane (DCM) solvent, 25 mL of ethanol, and 0.15 g of 4-dimethylaminopyridine (DMAP) catalyst. The reactor was then placed in an ice bath and cooled to 0 °C in a nitrogen atmosphere. Further, 3 mL (33 mmol) of vinylsulfonyl chloride was added dropwise under an inert atmosphere to the reaction mixture, followed by the addition of 20 mol % of triethylamine, and the mixture was allowed to stir for 4 h at 0 °C using an ice bath. The evolved HCl gas was trapped in an aqueous Na_2_CO_3_ scrubber, forming a NaCl solution, which was reused in washing steps. Further, 5 g of dried lignin and 35 mL of dimethyl sulfoxide (DMSO) were charged into the reactor. The reaction mixture was allowed to stir at 40 °C for 6 h in the presence of the DMAP catalyst. The mixture was allowed to cool to room temperature. To separate the vinyl ester sulfonated lignin macromonomer, the reaction mixture was added to cold, acidified distilled water (pH ∼3, adjusted with dilute HCl) under vigorous stirring. The filtered product was then dissolved in 50 mL of ethyl acetate, and the obtained organic solution was dried using anhydrous sodium sulfate, filtered, and concentrated under reduced pressure to obtain the purified vinyl-functionalized lignin, and the recovered DMSO was kept for recycling. The transesterification reaction mechanism of ethyl vinyl sulfonate with lignin is demonstrated in Figure 1.

**Figure 1:**
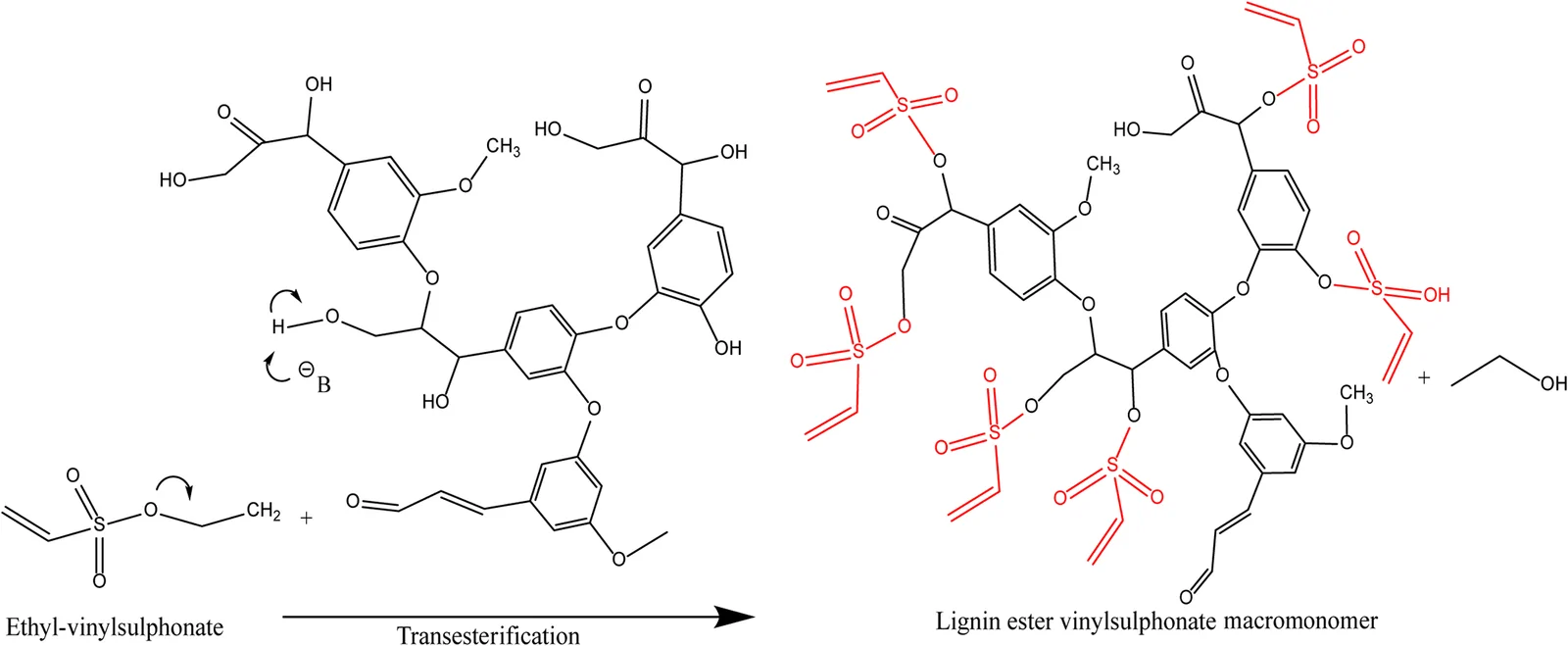
Proposed reaction mechanism for the transesterification of ethyl vinyl sulfonate with lignin.

### 2.4 Synthesis of the Green Chitin-*g*-Poly­(VESL/VIm)/CaCO_3_/Glycerol Self-Healing, Thermoresponsive Nanocomposite Hydrogel

After the synthesis of the VESL monomer, a typical free radical polymerization procedure was conducted in a four-neck 250 mL reactor equipped with a condenser, thermometer, mechanical stirrer, and nitrogen source. The designed amount of chitin and CaCO_3_ nanoparticles was added to 100 mL of distilled water (according to the wt % of each run) and stirred at a low rate until a clear suspension was obtained. To the reaction mixture, the designed amounts of the synthesized VESL and VIm were added to the reaction mixture (according to the specified mole ratio of each run). The reaction mixture was heated to the designed reaction temperature, and potassium persulfate and glycerol as a physical cross-linker were charged to the reactor. The copolymerization reaction continued under a nitrogen atmosphere for 12 h. The resulting hydrogel was further washed with acetone (4 × 50 mL). The resulting hydrogel was further dried at 60 °C for 24 h. The final polymerization yield was determined gravimetrically to be 96.8%. Table 1 summarizes the brine formulations utilized during subsequent characterization. Finally, the synthesized chitin-*g*-poly­(VESL/VIm)/CaCO_3_/glycerol nanocomposite hydrogel was engrossed into a 350,000 ppm brine solution for 24 h to attain maximum swelling. The swelling ratio of the synthesized hydrogel was further determined using eq 1. The synthetic scheme of the synthesized chitin-g-poly (VESL/VIm)/CaCO_3_/glycerol self-healing, thermo-responsive hydrogel is presented in Figure 2.

**Table 1:** Formulations of the Brine Mixtures Utilized during Rheological Characterization

	Ions				
Total dissolved solids (TDS)	10	40	100	150	350
Na^+^ ion (NaCl), g L^–1^	9.32	37.31	93.28	139.92	326.48
K^+^ ion (KCl), g L^–1^	0.074	0.29	0.74	1.11	2.59
Mg^2+^ ion (MgCl_2_.6H_2_O), g L^–1^	0.15	0.60	1.5	2.25	5.25
Ca^2+^ ion (CaCl_2_.2H_2_O), g L^–1^	0.44	1.79	4.48	6.72	15.68

**Figure 2:**
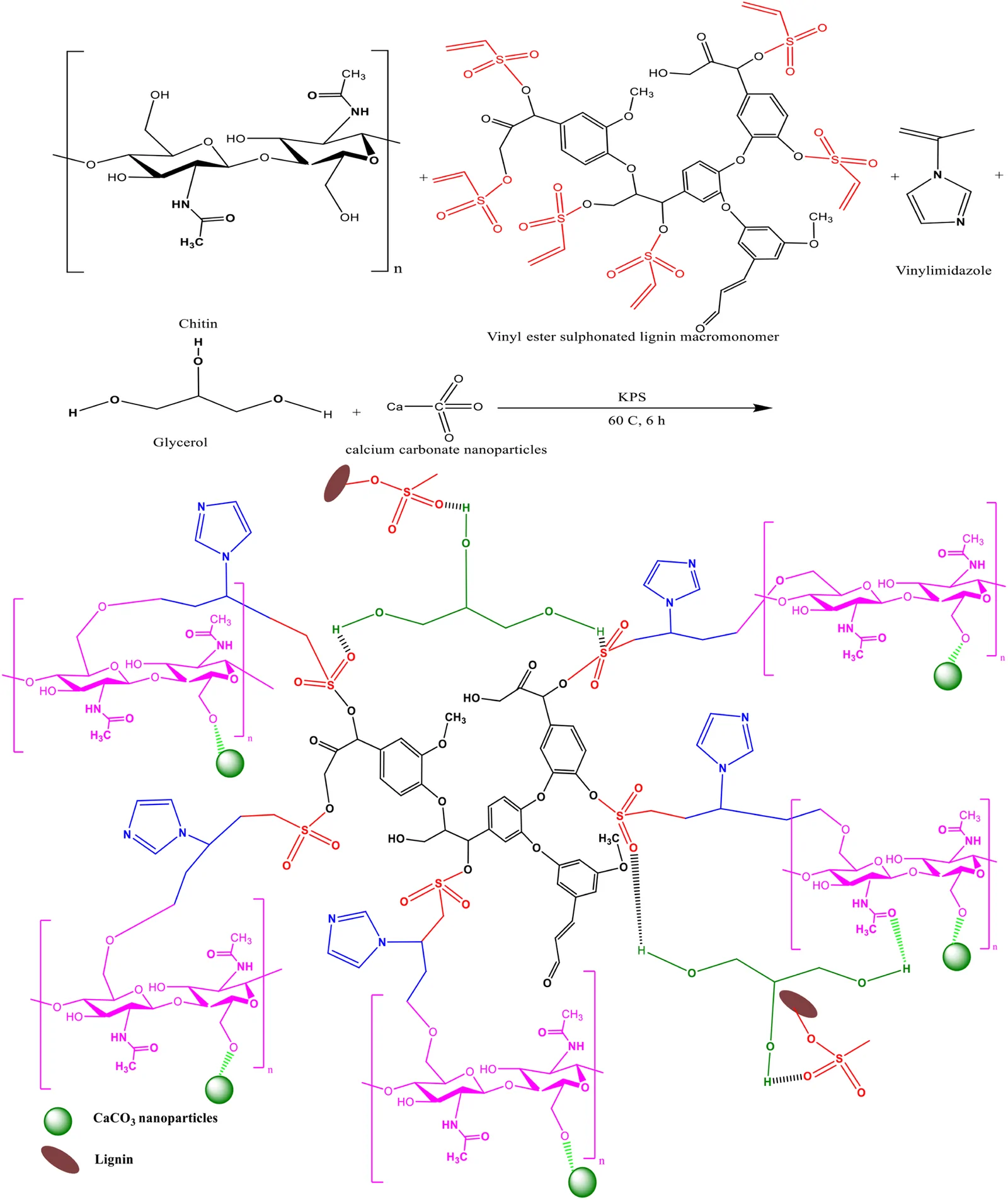
Synthetic scheme of the chitin-*g*-poly­(VESL/VIm)/CaCO_3_/glycerol self-healing, thermoresponsive hydrogel.

### 2.5 Brine Formulations

Double-distilled water was utilized to formulate brine solutions with different salinities. The specific chemical compositions of saline mixtures used in the measurements of the swelling ratio and storage modulus and flooding experiments are indicated in Table 1.

### 2.6 Characterization Techniques

Infrared spectrum analysis was accomplished using an FT-IR spectrometer (Shimadzu FTIR-8400). The ^1^H NMR analysis of the synthesized VESL macromonomer and the self-healing, thermoresponsive nanocomposite hydrogel was performed using a Bruker Avance 400 spectrometer (Bruker, Rheinstetten, Germany). Thermal stability was investigated under a nitrogen atmosphere using a Shimadzu TGA-50 thermogravimetric analyzer, ramping the temperature from 25 to 900 °C at a steady heating rate of 10 °C min^–1^. A Field-Emission Scanning Electron Microscope (Hitachi S-4800 II Model) was utilized to examine the hydrogel morphology following sample preparation with an E-1010 sputter coater on freeze-dried specimens. Elemental analysis of the synthesized nanocomposite hydrogel was performed by an Energy-Dispersive X-ray spectrometer equipped with an EDAX Octane Pro detector. The storage modulus of the swollen synthesized nanocomposites was evaluated using an Anton Paar MCR 302 rheometer equipped with a cone and a 25 mm diameter plate and 2 mm spacing, equipped with a solvent trap. The synthetic brine with varying salt concentrations ranged from 10,000 to 350,000 ppm.

### 2.7 Swelling Ratio Measurement

The swelling ratio was measured gravimetrically by soaking dry hydrogel samples (e.g., 0.5 g) in 100 mL of brine solutions under controlled temperatures (25–160 °C) for designated time intervals. The hydrogel samples were left soaking in brine under stirring for 24 h to ensure complete contact between all the particles and the brine solution. The swollen hydrogel samples were filtered from the saline solution and then weighed to calculate the swelling ratio (g/g) using eq 1.

1 $$SR=\left(\frac{W_{s}-W_{d}}{W_{d}}\right)$$

where *W*_*s*_ is the weight of the swollen sample and *W*_*d*_ is the weight of the dry hydrogel sample before swelling. The measurements were conducted three times, and the average values were calculated. The composition of saline solutions used in this study is indicated in Table 1.

### 2.8 Core-Flooding Experiments

Core displacement tests were carried out using a flooding setup designed to mimic the flooding process under reservoir conditions, as indicated in Figure 3. This setup included two accumulators filled with hydrogel solution and brine, along with a Bronkhorst EL-PRESS pressure meter and a Research Limited model 260D syringe pump. The experiment began with core preparation, where a dried standard carbonate core was weighed, and its dimensions were recorded, as detailed in Table 2. The core was further saturated with synthetic brine (350,000 ppm) under vacuum.^88,89^ The saturated core was further placed inside a core holder set to 500 psi and 160 °C, which was connected to the accumulators. Brine was injected at different flow rates (10, 8, 6, and 3 mL/min), and the pressure differential was recorded to calculate absolute permeability using Darcy’s law. Further, crude oil was injected until the water cut dropped below 2.0%. Then, brine was injected until pressure gradients were recorded until the water cut reached 98%. The water relative permeability was evaluated at the residual oil saturation (S_or_). The synthesized hydrogel was further injected into the core at several pore volumes. The relative permeabilities to water and oil after hydrogel flooding were determined to evaluate the effect of the hydrogel. The effectiveness of the gel in reducing water permeability, in comparison to oil permeability, was determined by calculating the Residual Resistance Factor (RRF), following the approach reported by Mahran et al.^90,91^ and using eq 2. This factor quantifies the extent to which polymer gel adsorption reduces permeability. The RRF could be evaluated from laboratory data, using the following equation, assuming that water viscosity is the same before and after hydrogel injection.

$$\mathrm{RRF}=\left(\frac{K_{W}}{K_{wp}}\right)=\left(\frac{\Delta P_{wp}}{\Delta P_{w}}\right)$$ 2

**Table 2:** Properties of Carbonate Cores Utilized in Flooding Experiments

Core sample	Diameter, cm	Length, cm	Bulk volume, cm^3^	Pore volume, cm^3^	Porosity, %	Brine permeability, mD
Core#1	5.081	11.52	233.58	48.1 ± 0.08	20.59 ± 0.5	50.25 ± 3.0
Core#2	5.081	11.52	233.585	47.85 ± 0.08	20.48 ± 0.5	50.13 ± 3.0
Core#3	5.081	11.72	237.63	47.25 ± 0.1	19.88 ± 0.4	50.37 ± 3.0

**Figure 3:**
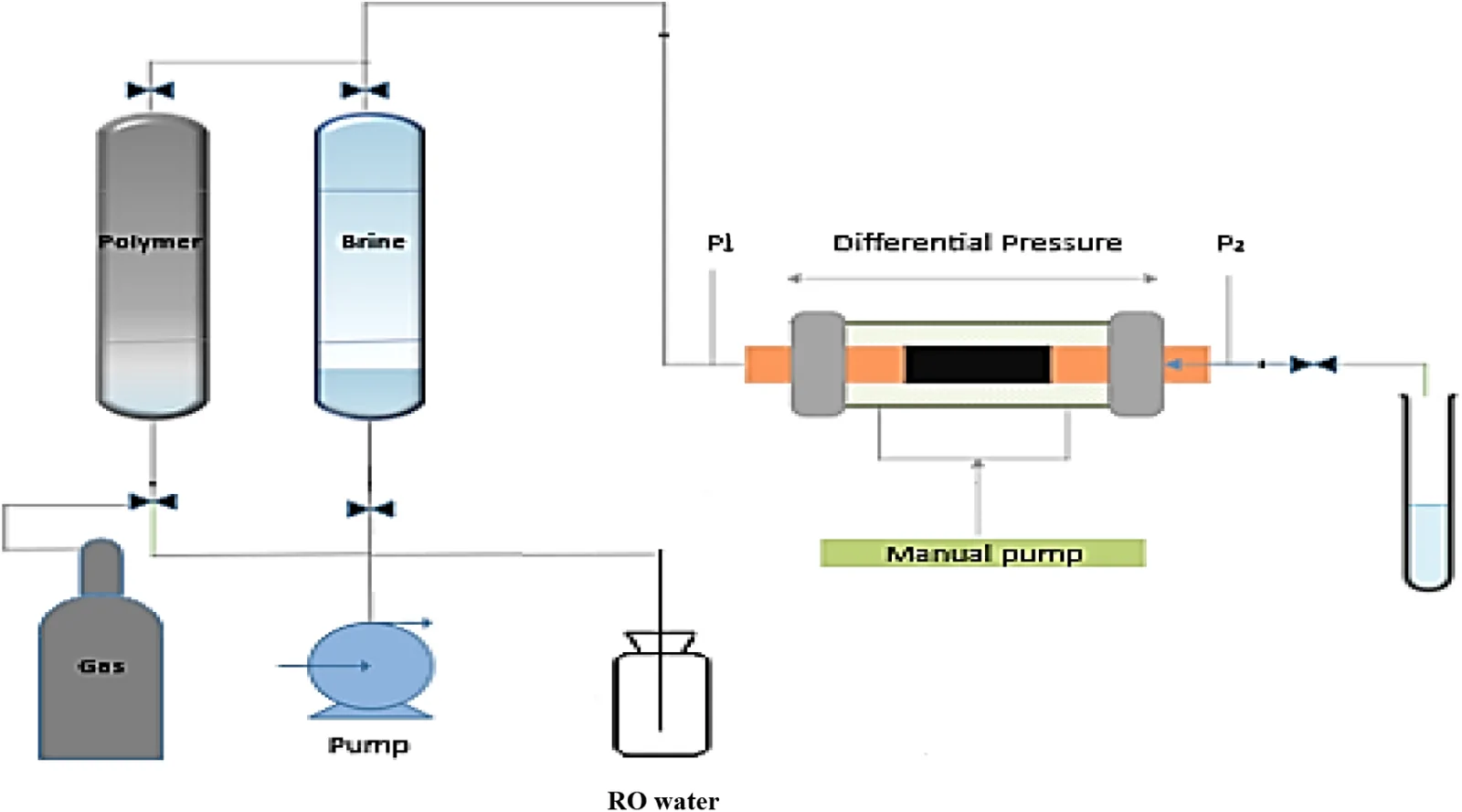
Schematic of the experimental core-flooding system.

### 2.9 Experimental Design

RSM was adopted as the design of experiments (DOE) approach to optimize the formulation of the self-healing, thermoresponsive nanocomposite hydrogel composition for a higher swelling ratio and storage modulus. The influence of four independent variables and their interactions on response variables (swelling ratio and storage modulus) was evaluated using RSM according to four factors and five levels of the CCD. In this research, the range of the defined variables was evaluated within five levels, coded as −2, −1, 0, 1, and 2, as indicated in Table 3. Based on the CCD, a 4-factor 5-level CCD was applied, with a total of 30 experiments performed in this research, as indicated in Table 4. The experimental runs were conducted in a randomized order to diminish the influence of unexplained inconsistency in the responses. The evaluated reaction variables were chitin concentration (*A*, wt %), VESL:VIm (*B*, molar ratio), CaCO_3_ loading (*C*, wt %), and glycerol concentration (*D*, wt %), while model responses were the swelling ratio of the synthesized nanocomposite (*Y*_1_) and the storage modulus (*Y*_2_, Pa).

**Table 3:** Experimental Design Variables and Their Coded Levels

		Levels				
Factor	Code	–2	–1	0	+1	+2
Chitin concentration (wt %)	A	5.18	10	32.5	55	59.8
VESL:VIm (molar ratio)	B	0.714	5	25	45	49.2
CaCO_3_ loading (wt %)	C	0.018	0.5	2.75	5	5.48
Glycerol concentration (wt %)	D	2.28	4	12	20	21.71

**Table 4:** Experimental Design Matrix with the Actual and Predicted Swelling Ratio and Storage Modulus Results.

Run	Chitin concentration, *A* (wt.%)	VESL:VIm, *B* (molar ratio)	CaCO_3_ loading, *C* (wt.%)	Glycerol concentration, *D* (wt.%)	Swelling ratio	Predicted swelling ratio	Storage modulus, G’ (Pa)	Predicted storage modulus, G’ (Pa)
1	55	45	0.5	4	35.33	33.84	1492	1503.91
2	10	5	0.5	20	36.66	33.96	200	206.57
3	55	5	0.5	4	41.66	39.33	750	742.24
4	59.82	25	2.75	12	50.00	49.98	1500	1545.94
5	32.5	25	2.75	12	73.33	73.86	1400	1380.98
6	55	45	0.5	20	30.00	26.12	1100	1061.53
7	5.18	25	2.75	12	75.00	82.02	550	591.22
8	10	45	0.5	4	66.66	66.77	400	358.63
9	55	45	5	20	23.33	27.16	1200	1252.99
10	10	5	5	20	26.66	27.66	10	19.030
11	10	45	5	4	83.33	81.14	500	541.08
12	32.50	25	2.75	2.28	60.00	63.32	1000	1070.33
13	32.50	25	2.75	12	76.66	73.86	1408	1380.98
14	32.50	0.71	2.75	12	53.33	56.04	800	844.15
15	55	45	5	4	45.00	45.63	2000	1940.37
16	10	45	0.5	20	76.66	73.30	352	367.25
17	32.50	25	2.75	12	83.33	73.86	1413	1380.98
18	55	5	5	20	10.00	7.81	458	446.31
19	10	45	5	20	76.66	76.92	350	304.70
20	55	5	0.50	20	15.00	16.69	400	379.86
21	32.50	25	2.75	21.71	43.33	47.01	690	706.82
22	32.50	25	2.75	12	73.33	73.86	1480	1380.98
23	10	5	5.00	4	45.00	46.80	190	175.41
24	32.50	25	0.018	12	45.00	57.67	1180	1258.02
25	32.50	25	5.48	12	66.66	61.00	1400	1409.14
26	32.50	25	2.75	12	76.66	73.86	1415	1380.98
27	32.50	25	2.75	12	77.50	73.86	1390	1380.98
28	55.00	5	5.00	4	38.33	41.20	1048	1053.69
29	32.50	49.2	2.75	12	78.33	82.62	1437	1480.00
30	10.00	5	0.50	4	46.66	42.35	150	117.95

### 2.10 Statistical Analysis

Regression analysis was achieved using a general quadratic polynomial equation to develop the model as indicated in eq 3, where *Y* is the predicted response (i.e., swelling ratio or storage modulus); *b*_0_, intercept coefficient constant; *b*_*i*_, *b*_*ii*_, and *b*_*ij*_, regression coefficients of linear, quadratic, and interactive terms, respectively; *x*_*i*_ and *x*_*j*_, model-independent variables (i ≠ j); and ε is the random error.

3 $$Y=b_{0}+\overset{n}{\underset{i=1}{\sum }}b_{i}x_{i}+\overset{n}{\underset{i=1}{\sum }}b_{ii}x_{i}^{2}+\overset{n-1}{\underset{i=1}{\sum }}\overset{n}{\underset{j>1}{\sum }}b_{ij}x_{i}x_{j}+\varepsilon$$

The predictive accuracy of the generated models was quantified through the coefficient of correlation (R^2^), adjusted coefficient of determination (R^2^adj), and predicted coefficient of determination metrics (R^2^pred). An analysis of variance (ANOVA) based on Fisher’s F-test was implemented to establish statistical significance at a 95% confidence level. The complete workflow, including the incorporation of DOE creation, regression modeling, visualization, and numerical response optimization, was carried out using Design-Expert version 12 (Stat-Ease Inc., Minneapolis, MN).

## 3 Results and Discussion

### 3.1 Development of Regression Model Equations

Design-Expert recommended the quadratic model for swelling ratio and storage modulus responses. Eqs 4 and 5 present the developed quadratic models and the empirical relationships linking the reaction variables to the studied responses.

4 $$Y_{1}=73.86-13.9A+10.94B+1.37C-6.71D-7.48AB-0.64AC-3.56AD+2.48BC+3.73BD-2.69CD-5.33A^{2}-3.07B^{2}-9.86C^{2}-12.68D^{2}$$

5 $$Y_{2}=1380.98+393.14A+261.84B+62.23C-149.69D+130.25AB+63.5AC-112.75AD+31.25BC-20.0BD-61.25CD-200.90A^{2}-148.48B^{2}-32.15C^{2}-333.99D^{2}$$

where *Y*_1_ and *Y*_2_ represent the swelling ratio and storage modulus, respectively, while *A*, *B*, *C*, and *D* represent the process variables of chitin concentration, VESL:VIm, CaCO_3_ loading, and glycerol concentration, respectively.

The regression models illustrate the impact of the reaction parameters on each response, where positive signs denote synergistic influences and negative signs denote antagonistic effects.^92,93^ The linear coefficients represent the independent effect of a single factor on the response, whereas the interaction coefficients describe the joint effects of the experimental variables. The quadratic coefficient denotes the influence of the excess of the variable on the response. As indicated in eq 4, chitin concentration and glycerol concentration have a negative effect on the swelling ratio, where increasing these variables has a decreasing influence on the swelling ratio. However, in eq 5, the positive signs across linear coefficients of A, B, and C demonstrate that increasing parameters such as chitin concentration, VESL:VIm, and CaCO_3_ loading directly increases the model response. It can be observed in eqs 4 and 5 that variation in chitin concentration has the strongest influence on both the swelling ratio and storage modulus, with the largest coefficient among other variables.

### 3.2 Model Adequacy Checking

The adequacy of the developed models was evaluated to define any errors related to the normal assumptions. Different approaches have been implemented to evaluate the adequacy of the predicted models. The coefficient of correlation (R^2^) assesses the adequacy of the predicted model, where an R^2^ closer to unity signifies a low difference between the predicted values of the model and the actual experimental values. The values of R^2^, R^2^adj, and R^2^pred have been assessed for the swelling ratio as 0.9913, 0.9831, and 0.9543, respectively. Furthermore, these parameters have been evaluated for storage modulus as 0.99, 0.981, and 0.941, respectively. Adequacy precision values have been assessed as 44.77 and 22.79 for models that represent swelling ratio and storage modulus, respectively. These results signify that the predicted models can be utilized to predict the design space. Statistical data obtained from variance analysis have been utilized to evaluate the significance of the predicted models. Furthermore, the significance of the model variables along with their interactions was also evaluated. The parameters from ANOVA are summarized in Tables 5 and 6.

**Table 5:** Analysis of Variance (ANOVA) for the Swelling Ratio Developed Model^a^

Source	Sum of squares	df	Mean square	F-value	p-value	Significance
Model	13145.44	14	938.96	27.54	<0.0001	HS
*A*—Chitin concentration	3298.97	1	3298.97	96.77	<0.0001	HS
*B*—VESL:VIm	2269.09	1	2269.09	66.56	<0.0001	HS
*C*—CaCO_3_ loading	35.61	1	35.61	1.04	0.3230	NS
*D*—Glycerol concentration	854.37	1	854.37	25.06	0.0002	HS
*AB*	895.01	1	895.01	26.25	0.0001	HS
*AC*	6.67	1	6.67	0.1958	0.6645	NS
*AD*	203.06	1	203.06	5.96	0.0275	S
*BC*	98.34	1	98.34	2.88	0.1101	NS
*BD*	222.51	1	222.51	6.53	0.0220	S
*CD*	115.56	1	115.56	3.39	0.0855	NS
*A* ^2^	153.98	1	153.98	4.52	0.0506	NS
*B* ^2^	51.10	1	51.10	1.50	0.2397	NS
*C* ^2^	525.77	1	525.77	15.42	0.0013	S
*D* ^2^	870.54	1	870.54	25.54	0.0001	S
Residual	511.36	15	34.09			
Lack of fit	444.12	10	44.41	3.30	0.0997	NS
Pure error	67.25	5	13.45			
Cor total	13656.80	29				

a where HS: highly significant, S: significant, and NS: not significant.

**Table 6:** Analysis of Variance for the Developed Storage Modulus Model^a^

Source	Sum of Squares	df	Mean square	F-value	p-value	
Model	8.256 × 10^6^	14	5.897 × 10^5^	184.89	<0.0001	significant
*A*—Chitin concentration	2.929 × 10^6^	1	2.929 × 10^6^	918.29	<0.0001	HS
*B*—VESL:VIm	1.299 × 10^6^	1	1.299 × 10^6^	407.33	<0.0001	HS
*C*—CaCO_3_ loading	73374.17	1	73374.17	23.01	0.0002	S
*D* —Glycerol concentration	4.246 × 10^5^	1	4.246 × 10^5^	133.13	<0.0001	HS
*AB*	2.714 × 10^5^	1	2.714 × 10^5^	85.11	<0.0001	HS
*AC*	64516.00	1	64516.00	20.23	0.0004	S
*AD*	2.034 × 10^5^	1	2.034 × 10^5^	63.78	<0.0001	HS
*BC*	15625.00	1	15625.00	4.90	0.0428	S
*BD*	6400.00	1	6400.00	2.01	0.1770	NS
*CD*	60025.00	1	60025.00	18.82	0.0006	S
*A* ^2^	2.430 × 10^5^	1	2.430 × 10^5^	76.21	<0.0001	HS
*B* ^2^	1.193 × 10^5^	1	1.193 × 10^5^	37.42	<0.0001	HS
*C* ^2^	5595.13	1	5595.13	1.75	0.2052	NS
*D* ^2^	6.038 × 10^5^	1	6.038 × 10^5^	189.32	<0.0001	HS
Residual	47839.76	15	3189.32			
Lack of fit	42754.43	10	4275.44	4.20	0.0633	NS
Pure error	5085.33	5	1017.07			
Cor total	8.303 × 10^6^	29				

a where HS: highly significant, S: significant, and NS: not significant.

The significance of the developed model has been assessed according to both the p-value and the F-test at the 95% confidence level. A p-value less than 0.05 indicates the significance of the corresponding parameter. The ANOVA results in Tables 5–6 demonstrate that both models are highly significant, each yielding a p-value below 0.0001. This statistical evidence confirms that the developed models reliably represent the experimental data. Furthermore, a lack-of-fit analysis was applied as an additional ANOVA measure to determine whether the models fail to accurately capture any variance within the experimental results. The lack-of-fit values for the swelling ratio and storage modulus models were reported as 0.0997 and 0.0633, respectively. The nonsignificance of the lack-of-fit values indicated that the developed models have successfully represented the experimental data.

Table 5 indicates that chitin concentration (*A*, wt.%), VESL:VIm (*B*, mole ratio), and glycerol concentration (*D*, wt.%) demonstrated a highly significant effect on the swelling ratio response, whereas CaCO_3_ loading (*C*, wt.%) demonstrated a nonsignificant influence on the swelling ratio with a p-value of 0.323. The ANOVA also indicated that there is a significant effect on the swelling ratio for the interaction of chitin concentration–VESL:VIm (*AB*), chitin concentration–glycerol concentration (*AD*), and VESL:VIm–glycerol concentration (*BD*). In contrast, the interactions between chitin concentration and CaCO_3_ loading (*AC*, p = 0.6645) and VESL:VIm and CaCO_3_ loading (*BC*, p = 0.1101) and the interaction of CaCO_3_ loading with glycerol concentration (*CD*, p = 0.0855) showed no significant effect on the swelling ratio. According to Table 6, chitin concentration (*A*, wt.%), VESL:VIm (*B*, mole ratio), and glycerol concentration (*D*, wt.%) demonstrated a highly significant effect on the storage modulus, whereas the loading of CaCO_3_ (*C*, wt.%) had a significant effect. Among the interactions of the two factors, almost all interactions significantly impacted the responses, with *AB* and *AD* showing highly significant effects (*p* < 0.0001), while *AC* (p = 0.0004), *BC* (p = 0.0428), and *CD* (p = 0.0006) were deemed significant. The only interaction factor that did not significantly affect the storage modulus was the interaction between VESL:VIm and glycerol concentration (*BD*, p = 0.1770). To validate the predicted models, the underlying ANOVA assumptions, specifically the normality of residuals, homoscedasticity of residuals, and randomness of errors, were evaluated.^92^ Homoscedasticity was evaluated using a plot of residuals versus predicted values. The consistent range of residuals across all levels confirms that the variable meets the homoscedasticity assumption, as illustrated in Figure 4 (a and b). Additionally, the randomness of errors was evaluated using a plot of residuals against the actual response values. As shown in Figure 4 (a and b), the residuals are randomly scattered without any discernible pattern, supporting the validity of the third ANOVA assumption. Furthermore, Figure 4 (c and d) indicates the experimental results versus predicted values by the developed models for both swelling ratio and storage modulus, respectively. The close alignment between the actual and predicted values confirms the precision of the model in predicting the model’s response.

**Figure 4:**
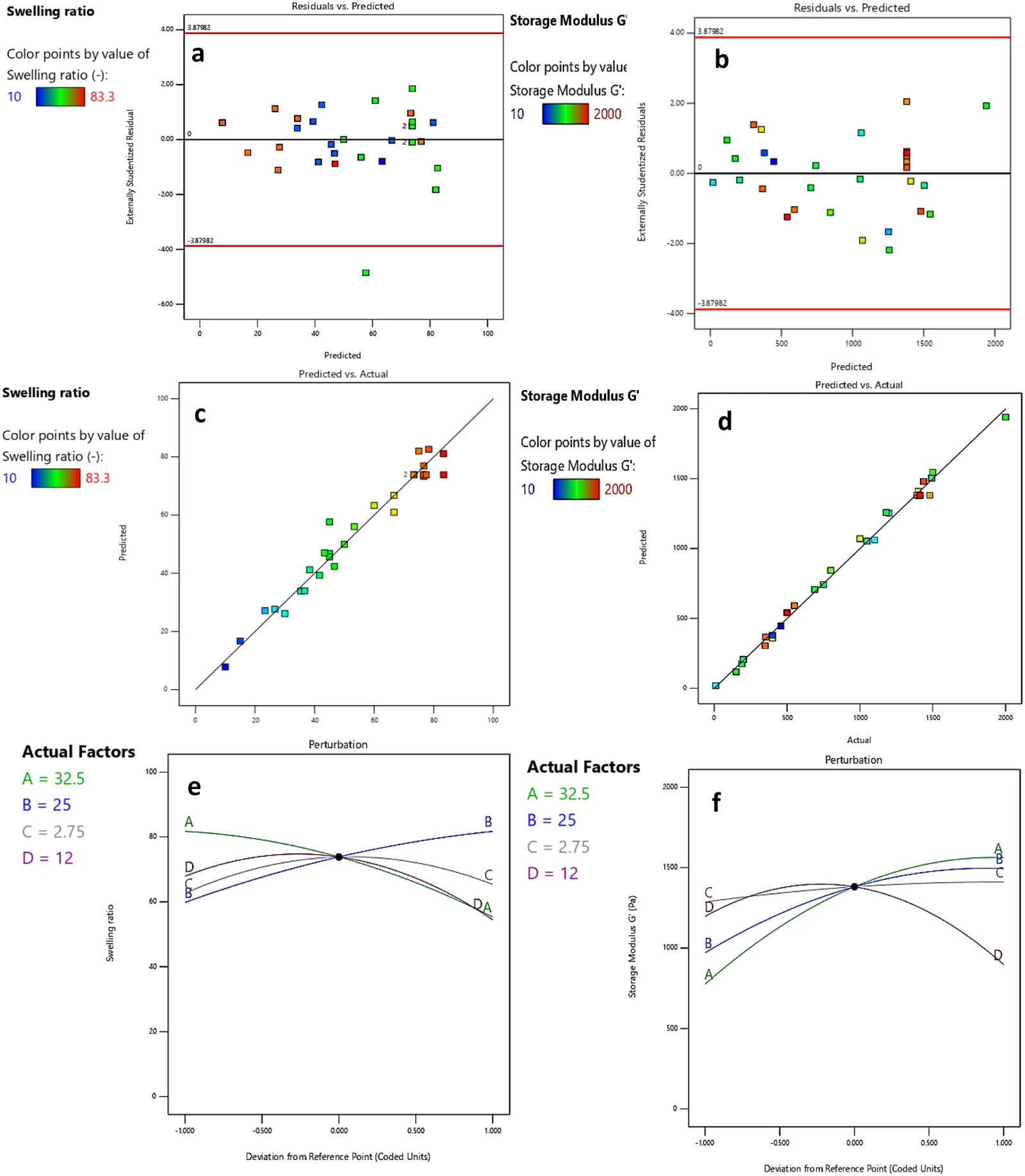
(a and b) Residuals versus predicted values for swelling ratio model and storage modulus models; (c and d) predicted versus actual values for swelling ratio and storage modulus models; (e and f) perturbation plot of swelling ratio and storage modulus models.

### 3.3 Effect of Process Variables and Their Interactions

This research has evaluated the swelling ratio and storage modulus of the chitin-*g*-poly­(VESL/VIm)/CaCO_3_/glycerol self-healing, thermoresponsive, nanocomposite hydrogel, synthesized with varying concentrations of chitin, VESL macromonomer, nanoparticle, and glycerol cross-linker. The performance of the hydrogel was evaluated under HTHS reservoir conditions of 350,000 ppm and 160 °C at a fixed nanocomposite hydrogel concentration of 0.1 wt %. This section presents an evaluation of the influence of the hydrogel composition on the swelling ratio and storage modulus of the synthesized nanocomposite hydrogel. Four process variables were investigated: chitin concentration, VESL:VIm ratio, and CaCO_3_ nanoparticle and glycerol concentration. The single and interactive effects of these variables on the swelling ratio and storage modulus have been discussed in this section.

#### 3.3.1 Effect of Chitin Concentration

In this research, the concentration of chitin incorporated in the hydrogel structure has a highly significant effect on both swelling ratio and storage modulus, as shown in Tables 5 and 6, respectively. As demonstrated in eqs 4 and 5, increasing chitin concentration has a negative effect on the swelling ratio of the synthesized hydrogel solutions, whereas it has a positive effect on G′ values evaluated at 350,000 ppm and 160 °C. As indicated in Figure 4 (e and f), the swelling ratio of the synthesized hydrogel decreases as the chitin content increases from 5 wt % to 55 wt %. However, G′ values significantly increased with increasing chitin concentration above 5 wt %. The decrease in swelling ratio values with increasing chitin content can be attributed to (i) the presence of −OH and −NH_2_ functional groups on chitin which promote hydrogen bonding with the polymer matrix, resulting in additional cross-linking of the hydrogel structure; (ii) these intramolecular interactions restrict the interaction between the polymer structure and the brine solution via hydrogen bonding, which hinders hydrogel expansion and water adsorption. Furthermore, these intramolecular interactions result in a reduction of hydrophilic groups, impairing the anchorage of water molecules. The increase in G′ values with increasing chitin concentration can be attributed to improved crystallinity and reduced chain mobility, which reduce the mesh size and restricts chain movement. The results are aligned with previous literature where the increase in polysaccharide concentration negatively affects the liquid uptake and swelling of the nanocomposite.^94,95^ The improvement in mechanical properties of chitin-based polymers with increasing incorporation of chitin has been previously reported by Carsi et al. and Liao et al.^94,95^

#### 3.3.2 Effect of the VESL:VIm Molar Ratio

Experimental runs have been conducted at VESL:VIm ratios between 2:1 and 10:1 to evaluate the effect of their change on the swelling ratio and storage modulus of the synthesized hydrogel. According to the ANOVA results summarized in Tables 5 and 6, the VESL:VIm molar ratio has a highly significant influence on the swelling ratio and storage modulus. The VESL:VIm molar ratio demonstrates a linear relationship with the swelling ratio and storage modulus, as ∼74 and 1500 Pa of the swelling ratio and storage modulus were reported at a higher VESL mol % of 45 mol %. As demonstrated in Figure 4 (e and f), swelling ratio and storage modulus values increase from 58.1 to 74 and 970 to 1500 Pa as the VESL:VIm molar ratio increased from 5% to 45%, respectively. Furthermore, the excess incorporation of the VESL:VIm molar ratio has a negative effect on the swelling ratio and storage modulus values, as shown in eqs 4 and 5, with a negative sign for the *B* ^2^ coefficient. The increase in the swelling ratio with the increase in the VESL:VIm molar ratio can be attributed to the presence of sulfonic acid groups along with the aromatic groups of lignin of the VESL macromonomer, which promotes binding of water molecules and improves hydration capacity through ion–dipole interactions in highly saline solutions. An increase in VESL mol % results in an increase in the network’s fixed negative charge density, which increases swelling through Donnan osmotic pressure. Despite the formation of hydrophobic aggregations of the aromatic domains contained in VESL, the incorporated sulfonic groups alter these domains into amphiphilic structures, where aromatic hydrophobic aggregations behave as a cross-linking site, whereas sulfonate-rich cores adsorb water. Furthermore, the amphoteric property of the nanocomposite hydrogel prevented dense ionic pairing at high sulfonation levels. The weakened hydrogen bonds and enhanced chain mobility with the increase of temperature also facilitated water uptake into the hydrogel. Sharker et al.^96^ reported similar phenomena. The increase of storage modulus with the increase of VESL can be attributed to the presence of both aromatic lignin compounds of the VESL macromonomer, which provide hydrophobic aggregation, acting as physical cross-linking sites, whereas sulfonate groups contribute ionic bridges with protonated VIm units. Furthermore, the amphoteric nature of the synthesized hydrogel reduced the free charge, which tightens the network and increases the storage modulus.

#### 3.3.3 Effect of CaCO_3_ Nanoparticle Concentration

The ANOVA results, summarized in Tables 5 and 6, demonstrate that the concentration of the CaCO_3_ nanoparticles, extracted from shrimp shells, has an insignificant influence on the swelling ratio of the synthesized nanocomposite, whereas it has a significant effect on the storage modulus values. The excessive increase in CaCO_3_ nanoparticle concentration has a negative effect on the swelling ratio of the synthesized nanocomposite, as indicated in eq 4 with a negative sign for the CaCO_3_ nanoparticle loading. The insignificant effect of excess CaCO_3_ nanoparticle loading on the swelling ratio values is demonstrated in Figure 4 (e and f), as the change in swelling ratio values at different levels of CaCO_3_ nanoparticle loading is negligible. This trend can be explained by the presence of CaCO_3_ in a nanosized structure, which improves the porosity and facilitates water uptake, whereas the release of Ca^2+^ ions neutralizes negatively charged groups, resulting in a controlled decrease in excessive swelling. The rigid nature of the CaCO_3_ nanoparticles behaves as reinforcement, which restricts the mobility of the polymer chain and, hence, increases the storage modulus at increased thermal stress. The improved modulus and controlled swelling ensure the structural integrity of the hydrogel without untimely collapse under HTHS reservoir conditions. Similar results were reported by Briggs et al.^97^ and Liu et al.^37^ They reported a similar behavior, with an insignificant effect of nanoparticle loading on the swelling ratio of the synthesized hydrogel.

#### 3.3.4 Effect of Glycerol Cross-Linker Concentration

The glycerol concentration has a highly significant influence on swelling ratio and storage modulus values, as reported in Tables 5 and 6, respectively. As demonstrated in Figure 4 (e and f), the glycerol concentration has an almost linear relationship with the swelling ratio within the range of 4 to 12 wt %, above which an increase in glycerol concentration has a negative effect on the swelling ratio. Under the free radical polymerization condition, glycerol acts as a physical cross-linker. The presence of multiple hydroxyl groups forms a dynamic intermolecular hydrogen bond with the functional domains of the graft copolymer. The increase in the swelling ratio with the increase of glycerol concentration is attributed to the highly hydrophilic nature of its hydroxyl groups, which enhance water affinity and expand the flexible network matrix, hence improving the swelling capacity.^98,99^ The flexible network promotes water absorption and retention within the polymer matrix. Beyond a glycerol concentration of 12 wt %, the synthesized hydrogel becomes saturated with cross-links, and the polymer network becomes too densely packed. Hence, adding more glycerol will negatively affect the swelling ratio. Similarly, the increase in glycerol concentration has indicated a positive influence on the storage modulus up to 12 wt %. However, the storage modulus decreased at higher glycerol concentration values (≤12 wt %). The decrease is attributed to over-cross-linking of the polymer chain, resulting in a rigid structure reducing their ability to deform or absorb stress, which reduces its storage modulus.^98,100,101^

### 3.4 Effect of Variable Interactions on the Response

Figure 5a indicates the three-dimensional response surface plot illustrating the interactive effects of chitin concentration wt % and VESL:VIm molar ratio, where CaCO_3_ nanoparticle loading and glycerol concentration were constant at their central midpoint values. As reported in the ANOVA results shown in Tables 5 and 6, the interactive effect of chitin concentration wt % and VESL:VIm molar ratio has a highly significant effect on the swelling ratio and storage modulus of the synthesized nanocomposites. These effects are related to the physical composition, network structure, and kinetics of polymerization at different levels of chitin concentrations. Figure 5a indicates that at low chitin concentration, the VESL:VIm molar ratio has a positive influence on the swelling ratio of the synthesized nanocomposite. This can be attributed to high incorporation of VESL macromonomer into the polymer structure at low chitin concentrations, which is facilitated by lower viscosity and improved chain mobility during the polymerization reaction. This allows a higher degree of polymerization and uniform copolymerization of VESL with the other existing monomers, including VIm. Furthermore, the presence of sulfonic acid along the VESL structure provides hydrophilic sites that improve water uptake inside the hydrogel structure. Conversely, at high levels of chitin concentration, the effect of VESL:VIm molar ratio is approximately negligible. This is due to the highly crystalline nature of chitin, which results in significant rigidity and cross-link density, reducing water absorption by the hydrogel. The rigid polymeric matrix increases the viscosity of the reaction medium, which hinders the diffusion of VESL macromonomer and radical propagation, leading to a lower degree of polymerization. Hence, the dehydration effect of the sulfonic group is minimized, and the swelling behavior is controlled by chitin’s restrictive network. However, the increase in VESL:VIm molar ratio at low chitin concentrations has a minimal influence on the storage modulus values, while at high chitin concentrations, it has a pronounced increasing effect on storage modulus values, as indicated in Figure 5b. At low chitin concentrations, the increment in the VESL:VIm ratio contributes to a slight increase in the modulus. The presence of loosely structured and flexible polymeric chains hinders the mechanical reinforcement, despite the presence of aromatic groups of VESL. Even with more efficient polymerization of VESL, the weak reinforcing support of the flexible matrix limits its ability to improve stiffness through hydrophobic aggregation of the macromonomer. However, increasing the VESL:VIm mole ratio in chitin-rich systems affords rigid support, where lower incorporation of VESL chains contributes to hydrophobic aggregations by physical entanglement. This results in a synergistic reinforcement effect where chitin provides structural rigidity and VESL and VIm monomers control the polymeric internal network through hydrophobic aggregations of lignin aromatic groupsand their amphoteric nature, which improves the storage modulus despite reduced swelling. These results are aligned with other literature.

**Figure 5:**
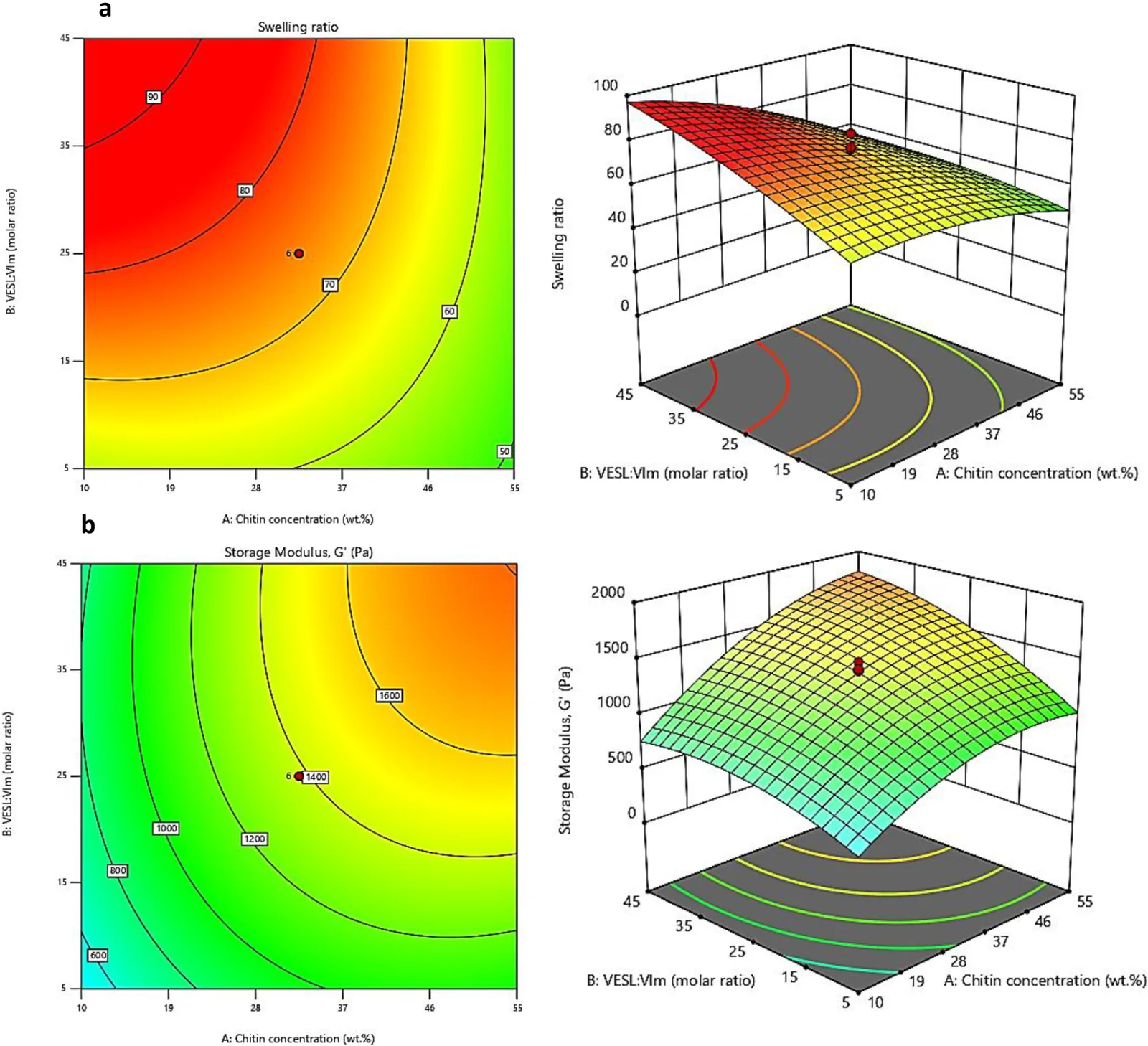
Three-dimensional and contour plots for the swelling ratio and storage modulus. The RSM plots were generated using the data shown in Table 4: (a) swelling ratio (%) as a function of VESL/VIm and chitin concentration, and (b) storage modulus (%) as a function of VESL/VIm and chitin concentration.

The interaction between chitin and glycerol concentrations has indicated a significant influence on the swelling ratio and a highly significant influence on the storage modulus of the synthesized nanocomposites, as summarized in Tables 5 and 6. The three-dimensional response surface plot between the chitin and glycerol concentrations is demonstrated in Figure 6a. The interaction is observed in Figure 6a, where the effect of glycerol concentration at a chitin concentration of 10% has a slight increasing effect on the swelling ratio of the synthesized nanocomposite until nearly 16 wt %, where the increase of glycerol concentration facilitates additional cross-link formation, leading to stabilization of the structural network of the hydrogel and improved swelling capacity owing to the formed balance between cross-link density and free volume, whereas further increase in glycerol concentration above 16 wt % results in a decrease in the response of the swelling ratio. The behavior is attributed to over-cross-linking, resulting in a decrease in the swelling ratio. Conversely, the effect of glycerol loading at a chitin concentration of 55 wt % is different, where there is a slight increase in the swelling ratio values until a glycerol concentration of 10 wt %, where a decrease in the response is observed as the glycerol concentration increases at a chitin concentration of 55 wt %. This indicates that glycerol concentration has a decreasing effect at higher values, yet the decreasing effect is more pronounced at a chitin concentration of 55 wt %. Furthermore, chitin concentration has a more significant decreasing influence on the swelling ratio of synthesized nanocomposites at higher levels of glycerol concentration.

**Figure 6:**
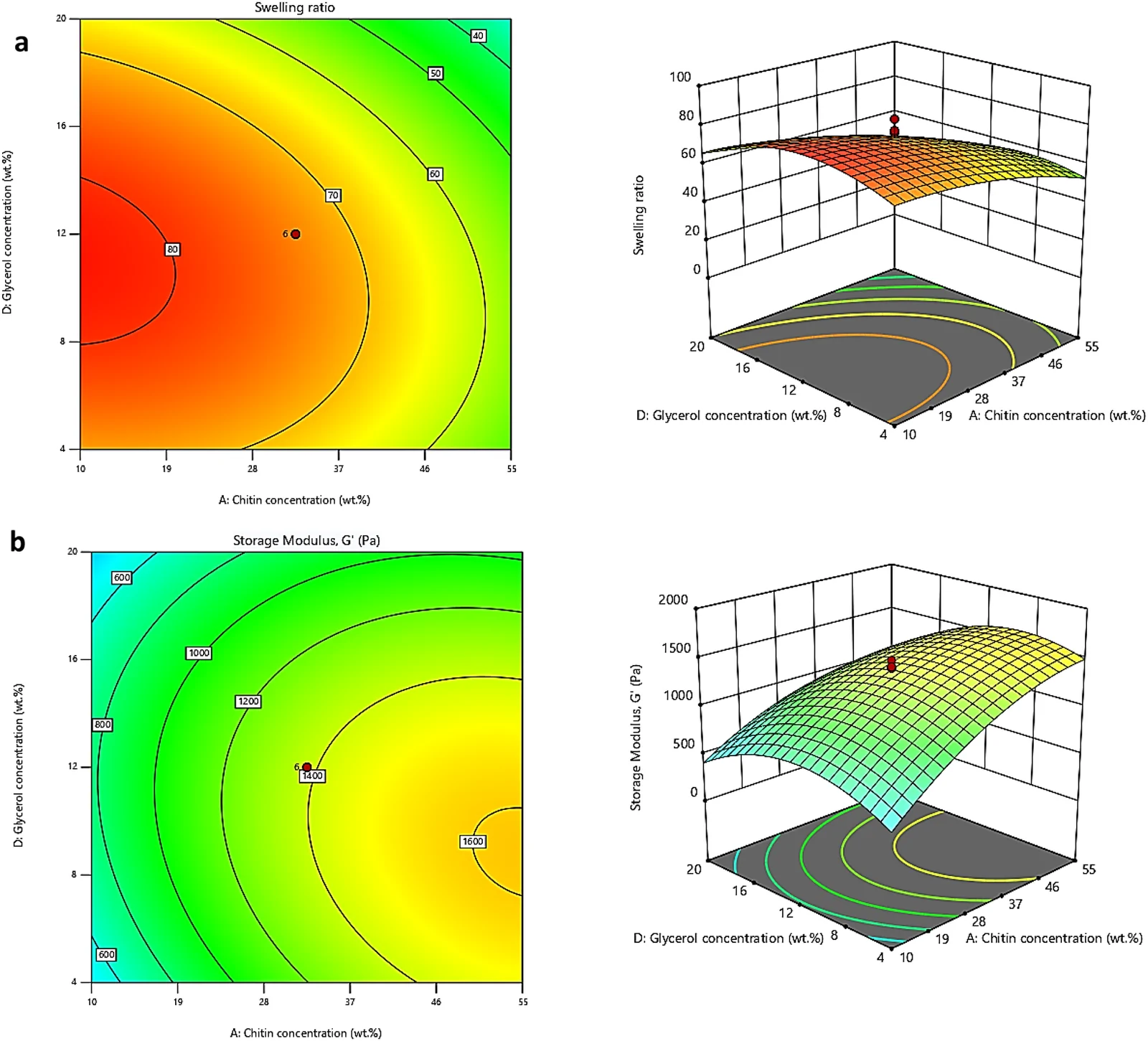
Three-dimensional and contour plots for the swelling ratio and storage modulus. The RSM plots were generated using the data shown in Table 4: (a) swelling ratio (%) as a function of glycerol and chitin concentrations, and (b) storage modulus (%) as a function of glycerol and chitin concentrations.

The interactive effect of chitin and glycerol concentrations on storage modulus is indicated in Figure 6b. As indicated in Figure 6b, at a chitin concentration of 5%, glycerol concentration has an increasing effect on the storage modulus until nearly 16 wt %, above which there is a slight decrease in storage modulus with the rise of glycerol concentration. The initial increase of the storage modulus with increasing glycerol concentration up to 16 wt % is attributed to the increment in the cross-link density and network cohesion of the synthesized hydrogel; however, excessive increase of glycerol concentration results in the formation of an over-cross-linked structure, which diminishes the structural uniformity of the hydrogel, resulting in a decrease in stiffness and its storage modulus. However, at a chitin concentration of 55 wt %, the storage modulus increases with glycerol concentration up to 12 wt %, after which a significant decrease occurs with further glycerol addition.The initial increase in the storage modulus is owing to enhanced load transfer between chitin and the glycerol-cross-linked domains. However, excessive glycerol concentration interrupts the hydrogel’s structural uniformity, leading to a substantial reduction in the mechanical strength and storage modulus. These results are in agreement with other literature, where the storage modulus and swelling behavior of hydrogels are controlled by the density of cross-link points and the interaction between the cross-linker and the polymer matrix.^100,101^

### 3.5 Optimization and Experimental Validation of Chitin-*g*-Poly­(VESL/VIm)/CaCO_3_/Glycerol Nanocomposite Hydrogel Composition

Design-Expert software was used for the optimization of the model responses (swelling ratio and storage modulus) through integrating the desirability of each model variable into a single value for the defined goals of the responses. The swelling ratio and storage modulus responses have been set to the maximum target along with the VESL:VIm molar ratio factor, whereas the rest of the formulation factors have been minimized as demonstrated in Table 7. Maximising the VESL:VIm ratio increases the content of VESL, a sustainable macromonomer, while decreasing the portion of VIm synthetic monomer and hence improving the sustainability and economic viability of the synthesized hydrogel. Minimizing the chitin, CaCO_3_ nanoparticles, and glycerol concentrations reduces the total used materials, while preserving self-healing ability and mechanical integrity.

**Table 7:** Optimization Constraints Are Used to Predict the Optimum Hydrogel Composition for Maximum Swelling Ratio and Storage Modulus

			Importance	Limits	
Factor	Code	Goal	Scale 1–5	lower	upper
Chitin concentration (wt %)	*A*	Minimize	4	10	55
VESL:VIm (molar ratio)	*B*	Maximize	4	5	45
CaCO_3_ loading (wt %)	*C*	Minimize	4	0.5	5
Glycerol concentration (wt %)	*D*	Minimize	4	4	20
Swelling ratio, g/g-	*Y*_1_	Maximize	5	50	85
Storage modulus, Pa	*Y*_2_	Maximize	5	1500	1900

Utilizing this simultaneous multiresponse desirability technique, the Design-Expert software evaluated the design space and generated 70 solutions with varying desirability. From these solutions, the formulation that maximized the desirability function was selected to balance the conflicting chemical properties of the formulation variables. In particular, maximizing the sustainable VESL content and implementing a strong structural chitin framework (35.8 wt %) establishes a foundation for network stability, while the careful selection of glycerol concentration (9.1 wt %) serves as the key adjusting variable that balances the inherent thermodynamic inverse relationships between swelling capacity and mechanical stiffness. As demonstrated in the individual and interactive trend analyses (Sections 3.3.4 and 3.4), insufficient glycerol levels do not stabilize the multicomponent network, whereas too high a concentration leads to over-cross-linking, which undermines structural uniformity, limits pore expansion, and diminishes storage modulus performance. By numerically optimizing the entire composition space simultaneously, the software identified the combination of 35.8 wt % chitin, a VESL/VIm ratio of 45, 3.5 wt % CaCO_3_, and 9.1 wt % glycerol as the optimum composition that maximizes structural strength without restricting water intake, yielding an optimized target pairing of predicted swelling ratio and storage modulus values of 78.2 and 1700 Pa, respectively, and maximum multi-response desirability function under optimum conditions. To validate the predicted optimum hydrogel composition, the swelling ratio and storage modulus of the hydrogel have been evaluated three times, and the results have been further averaged and considered as experimental results. The experimental validation has yielded a swelling ratio and storage modulus of 80.2 ± 1.5 and 1704 ± 12 Pa, respectively, which indicates the high accuracy of the models, demonstrating low relative errors of only 2.56% and 0.24% for the swelling ratio and storage modulus, respectively.

### 3.6 Spectroscopic Analysis and Molecular Weight Estimation

The FTIR spectra of the VESL macromonomer (Figure 7a) indicate characteristic absorption peaks at 3329 cm^–1^ that were attributed to the hydroxyl group (OH), and absorption peaks at 2851.91 cm^–1^ and 2920.30 cm^–1^ are assigned to the C–H stretching vibration of the methoxyl groups of lignin aromatic groups. The presence of the characteristic peaks of conjugated carbon–carbon double bonds (C=C) is also found at 1596 cm^–1^ and 1511 cm^–1^. The peaks at 1210, 1168, and 1122 cm^–1^ are attributed to C–O– and O=C–O– stretching vibrations owing to the presence of methoxy, ester, or ether functional groups. The presence of absorption peaks at 1123.2 cm^–1^, 1044.5 cm^–1^, 612 cm^–1^, and 511 cm^–1^ of S–C, O–S–, and S–O groups and 1630 cm^–1^ of the vinyl group confirms the incorporation of the group and a successful synthesis of the VESL monomer.

**Figure 7:**
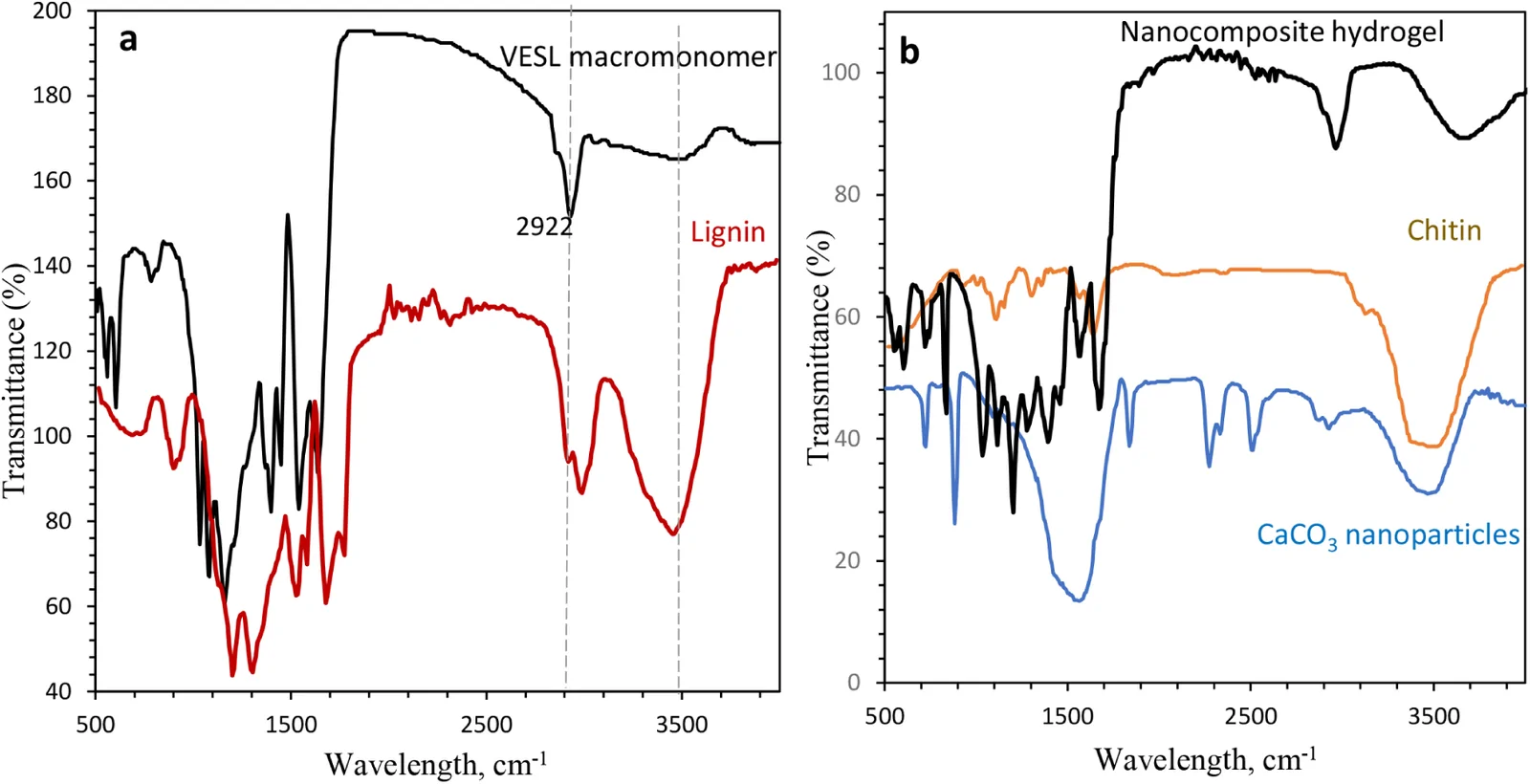
Fourier Transform Infrared (FTIR) spectroscopy of (a) lignin and VESL macromonomer and (b) chitin, CaCO_3_ nanocomposite, and chitin-*g*-poly­(VESL/VIm)/CaCO_3_/glycerol nanocomposite hydrogel.

The FTIR spectra of chitin and chitin-*g*-poly­(VESL/VIm)/CaCO_3_/glycerol nanocomposite hydrogel are presented in Figure 7b. The FT-IR spectrum of chitin demonstrates absorption peaks at 1650 and 1550 cm^–1^, which were attributed to stretching vibrations of C=O and the bending vibration of N–H coupled with C–N stretching vibrations,^35^ respectively, signifying the presence of CONH_2_ groups. The characteristic peaks at 3400–3500 cm^–1^ are attributed to the existence of −OH and N–H stretching vibrations,^102^ which indicates that the chitin hydrogel contains the hydroxyl active group.^103^ The characteristic peaks at 1160–900 cm^–1^ correspond to C–O–C and C–O of the pyranose ring system, alongside the etheric linkages intrinsic to the N-acetylglucosamine structural segments. FT-IR spectroscopy of CaCO_3_ demonstrates the presence of a strong absorption peak at 1440 cm^–1^, signifying the presence of the stretching vibration of C–O, and absorption peaks at 876 and 714 cm^–1^ assigned to the bending vibrations of C–O.^103,104^ FT-IR spectroscopy of the chitin-*g*-poly­(VESL/VIm)/CaCO_3_/glycerol hydrogel demonstrates that the characteristic peaks of C=N appeared at 1230 cm^–1^, which confirms the presence of VIm in the hydrogel composite. The characteristic absorption peaks of −NH_2_ at 3400 cm^–1^, carbonyl stretching at 1160–900 cm^–1^ of the pyranose ring, and the linkage between the N-acetylglucosamine units, the doublet peaks at 1400–1200 cm^–1^, confirm the existence of chitin in the synthesized nanocomposite hydrogel. The absorption peaks of C–H of the methoxyl groups of the lignin aromatic groups at 2851 cm^–1^ and 2930 cm−1 ^105^ and the −SO_3_– group at 1123.2 cm^–1^, 1044.5 cm^–1^, 612 cm^–1^, and 511 cm^–1^ indicate the incorporation of aromatic groups of lignin and the sulfonic group of the VESL macromonomer. The absorption peaks at 876 and 714 cm^–1^ are assigned to the bending vibrations of C–O in CaCO_3_ nanoparticles.^103,104^ The disappearance of the vinyl group’s characteristic band between 1600 and 1650 cm^–1^ proves successful and complete polymerization. The average molecular weight of the extracted chitin was measured by Gel Permeation Chromatography (GPC) equipped with a Waters 515 HPLC pump and a Waters 2410 Refractive Index (RI) detector. The column temperature was set to 40 °C. The DMF salt solution was applied as a carrier eluent. The measurement was conducted at a flow rate of 1 mL/min. The measured molecular weight of chitin is 50,630 g/mol.

The chemical structure of the synthesized materials was proved using ^1^H NMR spectroscopy. ^1^H NMR (400 MHz, DMSO) of chitin, the synthesized VESL macromonomer and the nanocomposite hydrogel are demonstrated in Figure 8 (a–c). The ^1^H NMR plot of chitin indicated a singlet peak at δ ≈2.0 ppm attributed to the protons of the CH_3_ group of the N-acetyl group (−NHCOCH_3_) structure of chitin. The peaks at δ ≈4.6–4.7 ppm correspond to the anomeric proton (H-1) of the glycosidic linkages. The peaks between δ 3.3 and 4.1 ppm correspond to the H3–H6 ring protons.

**Figure 8:**
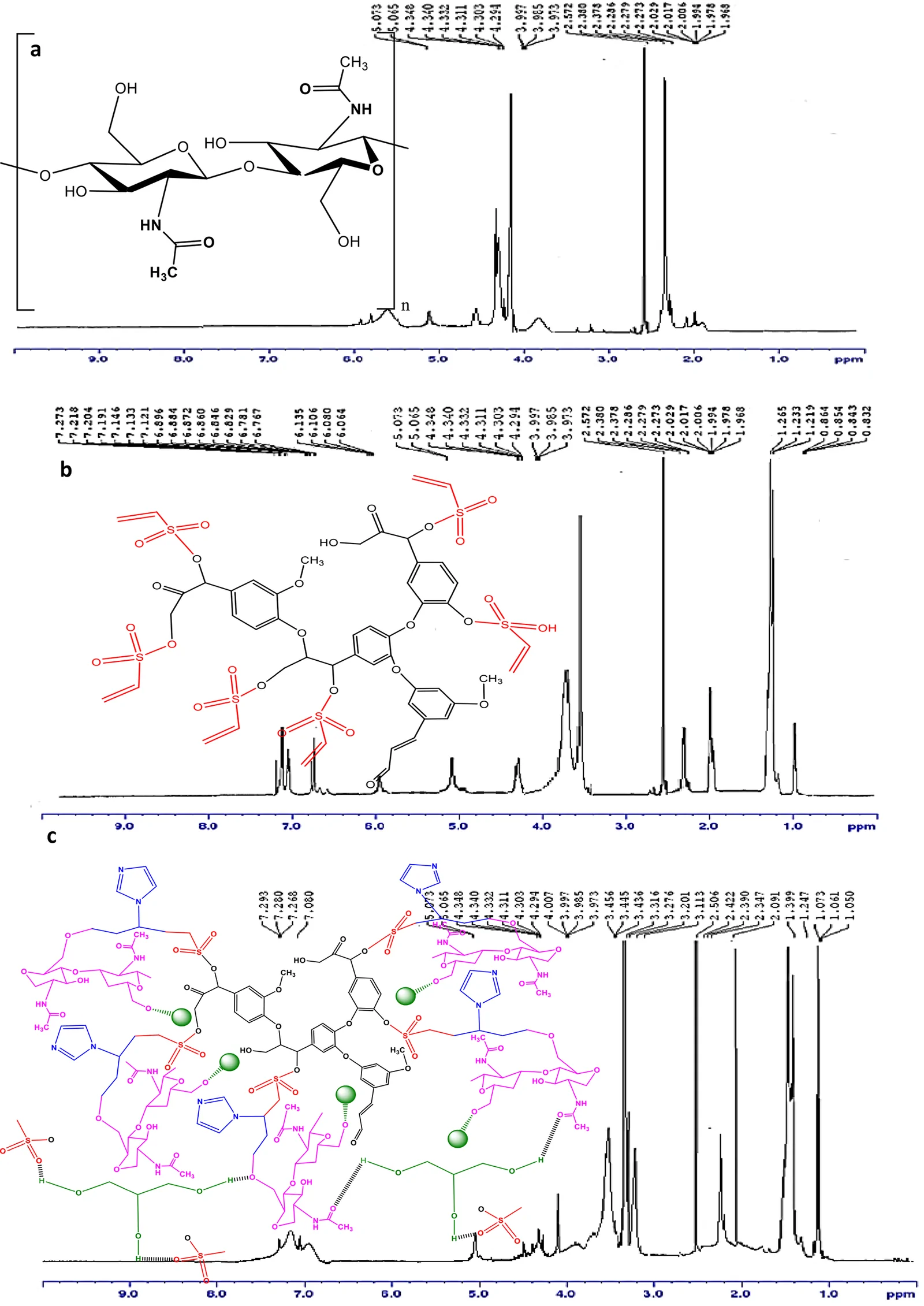
Proton ^1^H NMR spectra of (a) chitin, (b) VESL macromonomer, and (c) chitin-*g*-poly­(VESL/VIm)/CaCO_3_/glycerol nanocomposite hydrogel.

The ^1^H NMR spectra of the VESL macromonomer demonstrate that the characteristic chemical shifts at 1.6 ppm to 2 ppm can be attributed to aliphatic side-chain protons. The signal at 2.5 ppm is attributed to the residual DMSO-*d*_6_ solvent. The characteristic broad peak (3H singlet) at 3.5 ppm is assigned to the methoxy group (O=CH_3_) attached to the lignin aromatic ring. Aromatic protons of the lignin appear at δ (ppm) = 6.6–7.29, attributed to aromatic ring protons. The protons of the newly added vinyl sulfonate group are observed at 6.2–6.4 ppm for the protons (=CH) and 4.6–5.1 ppm for the terminal =CH_2_ group of the vinyl group. The presence of these vinyl groups proves the successful grafting of the vinyl sulfonate group and the successful synthesis of the VESL macromonomer.

The ^1^H NMR (400 MHz, DMSO-*d*_6_, 25 °C) spectrum of the hydrogel nanocomposite demonstrates a chemical shift at δ (ppm) = 1.39 ppm, which is assigned to the protons of the aliphatic CH_2_ groups, and the peak at δ = 2.09 ppm was assigned to the methyl (−CH_3_) of the N-acetyl group on chitin. The chemical shift between δ = 3.6–5.5 ppm corresponds to the anomeric and pyranose ring protons of chitin and the polymer backbone, and δ = 7.8 ppm corresponds to N-acetyl NH protons included in chitin. The signals between δ = 7.08–7.29 ppm correspond to the overlapped signals of the aromatic protons at the phenyl ring of the units and the imidazole ring protons (s, ^1^H, −NCHN−) of grafted VIm. These results prove the successful synthesis of the nanocomposite hydrogel owing to the absence of characteristic peaks of −CH_2_=CH– of VESL and VIm groups. Table 8 summarizes the spectroscopic characterization data of the VESL macromonomer and the chitin-*g*-poly­(VESL/VIm)/CaCO_3_/glycerol nanocomposite hydrogels.

**Table 8:** Spectroscopic Characterization Data of the VESL Macromonomer and Chitin-*g*-Poly­(VESL/VIm)/CaCO_3_/Glycerol Nanocomposite Hydrogel

	VESL macromonomer		Hydrogel nanocomposite	
Analysis	Frequency, cm^–1^	Assignments	Frequency, cm^–1^	Assignments
FTIR bands	3329	–OH	3410–3200	NH_2_ in the amide groups.
	2927	C–H, stretches =CH, CH_2_, alkene	2870–2930	(CH_2_, alkane)
	2850	CH_2_–, alkane	1123.2 cm^–1^, 1044.5 cm^–1^, 612 cm^–1^, and 511 cm^–1^	S–C
				O–S
				S–O
	1630	C=C, vinyl	1400–1200	C–O–C and C–N
	1560	C=C	1160–900	C=O, ester
	1511	C=C		
	1123	S–C	876 and 714 cm^–1^	C–O
	1044.5	O–S		
	612, 511	S–O		
	1210	C–C(O)–O, ester		
	1168	O=C–O–		
	1122			

	Chemical shift (δ) ppm	Chemical shift (δ) ppm
^1^H NMR	1.2–2 (aliphatic side-chain protons)	1.23–1.6 ( −CH_2_−)
	2.5 (2H, CH_2_–O–)	2.01 (H, CH−) methine group
	3.6–3.9 (3H, O=CH_3_) methoxy group	3.6–3.9 (3H, O=CH_3_)
		6.5–7.5 (5H, –CH=CH–) of benzene ring of lignin
	6.2–6.4 (1H, HCH=CH–SO_3_)	2.3 (3H, CH−) CH_3_ group of the N-acetyl group on chitin
	4.6–5.1 (2H, HCH=CH–S)	3.6–5.5 (H2–H6) chitin ring protons

	6.6–7.29 (d, 5H, –CH=CH–) at the aromatic ring	7.2 (s, 1H, −NCHN−) of VIm monomer



### 3.7 Thermogravimetric Analysis (TGA) and Derivative Thermogravimetric Analysis (DTG)

The thermal decomposition of chitin, chitin-poly­(VESL/VIm), chitin-poly­(VESL/VIm)/CaCO_3_, and chitin-poly­(VESL/VIm)/CaCO_3_/glycerol hydrogels was evaluated using TGA and DTG, as shown in Figure 9 (a and b). The TGA curve of the chitin hydrogel showed two main compositional stages. The initial weight loss up to ∼100 °C was attributed to water evaporation in the sample. The main thermal degradation stage was observed between 240 and 400 °C, demonstrating a sharp weight loss of ∼66%, which was attributed to the decomposition of the sugar ring and amide groups of the chitin structure and exhibited a maximum degradation temperature on the DTG curve of 280 °C. A final residual mass of 23% was retained at 950 °C.

**Figure 9:**
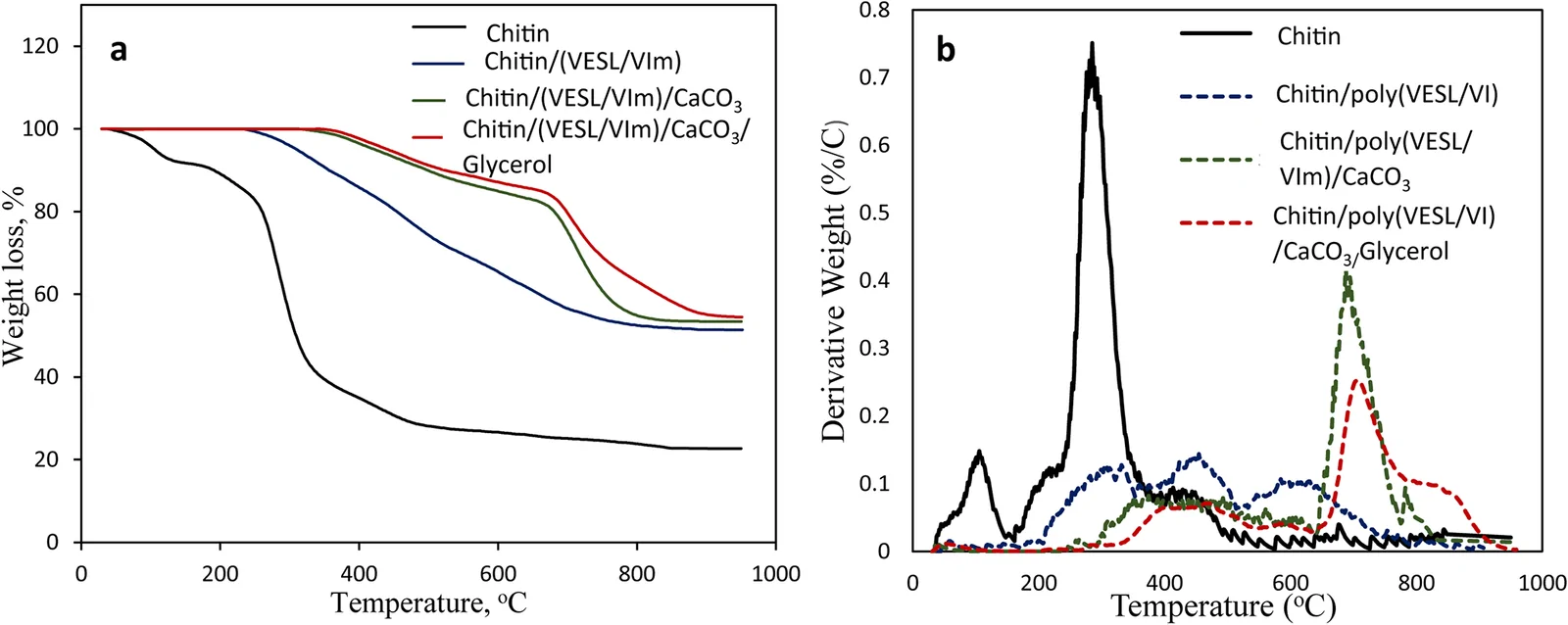
(a) Thermogravimetric analysis (TGA) and (b) derivative thermogravimetric analysis (DTG) of chitin, chitin-*g*-poly­(VESL/VIm), chitin-*g*-poly­(VESL/VIm)/CaCO_3_, and chitin-*g*-poly­(VESL/VIm)/CaCO_3_/glycerol nanocomposite hydrogel.

The TGA curve of the chitin-poly­(VESL/VIm) hydrogel demonstrated three decomposition steps above 200 °C. The initial thermal decomposition stage occurred at 200–400 °C with 16% weight loss, signifying the decomposition of the sulfonate (−SO_3_^–^) groups and β-O-4 linkages of VESL, which aligns with the thermal profile of lignosulfonates.^106,107^ The second thermal degradation stage was observed between 400 and 520 °C with a weight loss of 30%. This thermal degradation was due to the decomposition of the lignin aromatic ring, methoxy groups, and the breakage of C–H, C–C, N–O, and C–O bonds in the polymer chain.^108^ The last decomposition stage occurred between 520 and 800 °C due to further decomposition of the aromatic network and C–O–C or C=O bonds in lignin.^109^ The shift of the main degradation temperature to 495 °C demonstrates a considerable enhancement in thermal stability through incorporation of the VESL macromonomers.

The TGA curve of the chitin-poly­(VESL/VIm)/CaCO_3_ hydrogel showed an onset degradation temperature (T_10_) above 250 °C.^110^ The second decomposition stage occurred between 300 and 600 °C. This stage involved dehydration, decarboxylation, deamination, and depolymerization of the saccharide rings, amide, carboxyl groups, and the copolymers, respectively.^111^ The final decomposition stage occurred between 620 and 770 °C, with a maximum degradation temperature on the DTG curve of 700 °C, mainly attributed to the thermal calcination of CaCO_3_.^112^ A significant residual mass of 48% was retained at 750 °C, further confirming the high thermal stability imparted by CaCO_3_ particles.

The TGA curve of the chitin-poly­(VESL/VIm)/CaCO_3_/glycerol nanocomposite hydrogel also demonstrated three decomposition stages. The first decomposition stage reported a temperature of up to 340 °C, which was attributed to the evaporation of free water. The weight loss in the second stage (300–600 °C) resulted from the combined decomposition of the aromatic ring and methoxy groups of the VESL monomer, the sugar ring, and amide groups, along with the decomposition of the glycerol molecules included in the polymer chains.^108^ In the last stage between 600 and 760 °C, the DTG curve exhibited a maximum degradation rate at 715 °C, which is associated with the calcination of the embedded CaCO_3_ nanoparticles. The final mass residual of the chitin-poly­(VESL/VIm)/CaCO_3_/ glycerol and chitin was 55.24%, significantly higher than that of pure chitin (23%), clearly indicating the superior thermal stability of the synthesized nanocomposite network.

### 3.8 Morphological and Elemental Characterization

The surface morphology of different hydrogels synthesized with different compositions is indicated in Figure 10a–d to further evaluate the influence of monomer composition on the morphology of the hydrogel microstructure, ensuring the grafting and cross-linking processes of the synthesized hydrogels. A hydrogel solution of 0.1 wt % concentration was prepared at 350,000 ppm and 160 °C. Further, the hydrogel samples were frozen in liquid nitrogen and freeze-dried for SEM evaluation.

**Figure 10:**
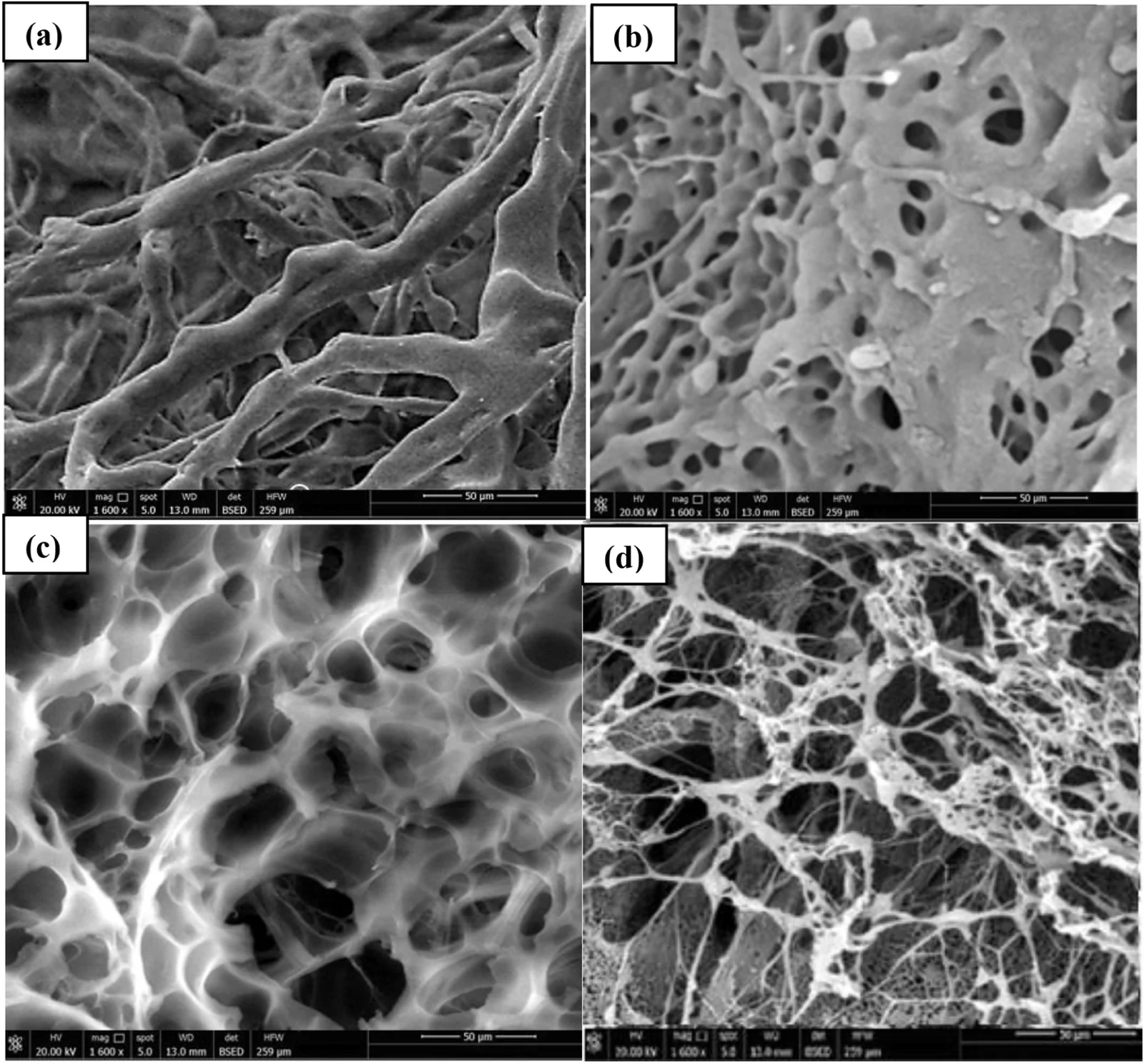
(a–d) SEM images of the 0.1 wt % hydrogel: (a) chitin, (b) chitin-*g*-poly­(VIm), (c) chitin-*g*-poly­(VESL/VIm), and (d) chitin/poly­(VESL/VIm)/CaCO_3_/glycerol nanocomposite hydrogel.

Figure 10a indicates the SEM image of chitin, where a fibrous surface with a dense, limited-porosity structure was observed. The absence of a three-dimensional hydrogel network able to swell in the chitin structure resulted in limited water retention; therefore, a fibrous, limited-porosity structure is observed. The SEM image of the chitin/poly­(VIm) hydrogel (Figure 10b) indicates the presence of a limited porous structure with small pores. In contrast, the SEM image of the chitin/poly­(VESL/VIm) hydrogel (Figure 10c) indicates a cross-linked porous structure with increased pore size compared to the chitin/poly­(VIm) hydrogel. The amphiphilic property of the VESL macromonomer, imparted by its hydrophobic aromatic group and hydrophilic sulfonic groups, improves the swelling property of the synthesized hydrogel, resulting in enhanced pore expansion. Figure 10d shows the SEM image of the chitin/poly­(VESL/VIm)/CaCO_3_/glycerol nanocomposite hydrogel. A massive number of intermolecular linkages and smooth pore walls, showing an ultrafine reticulated sponge-like structure consisting of highly interconnected nanofibrous fibrils, can be observed between the hydrogel chains following the integration of glycerol and CaCO_3_ nanoparticles. This dense conformation results in a significant increase in the hydrodynamic volume and consequently improves the mechanical properties of the nanocomposite. The cross-linking influence of glycerol creates strong hydrogen bonds, forming a robust three-dimensional network structure that resists pore collapse at high HTHS conditions. Furthermore, the embedded CaCO_3_ nanoparticles further strengthen the structural network of the hydrogel, whereas the incorporation of the VESL macromonomer provides amphiphilic properties to the synthesized hydrogel, which improves the swelling property alongside the mechanical integrity of the synthesized hydrogel. The interconnected sponge-like morphology demonstrates structural durability, high elasticity, and superior swelling properties of the synthesized hydrogel tailored for water shutoff applications in extreme environments.

Energy-dispersive X-ray (EDX) analysis of the synthesized chitin/poly­(VESL/VIm)/CaCO_3_/glycerol hydrogel nanocomposite was performed to identify the elemental composition of the synthesized hydrogel nanocomposite and to ensure the incorporation of CaCO_3_ nanoparticles embedded in the nanocomposite structure. The EDX spectrum demonstrates different peaks that are attributed to carbon (C) 55.77%, oxygen (O) 34.24%, nitrogen (N) 3.76%, sulfur (S) 6.23%, and calcium (Ca) 0.20% atoms. The existence of the Ca atom peak was easily detected. The EDX spectrum in Figure 11 demonstrated that carbon (C) and oxygen (O) are the constituent components of the hydrogel nanocomposite. The presence of C is attributed to the organic polymer backbones, while O elements are attributed to esters and sulfonate groups within the cross-linked network as proven from the chemical structure of the hydrogel. The calcium component is observed because of the encapsulation of CaCO_3_ nanoparticles in the polymer composite.

**Figure 11:**
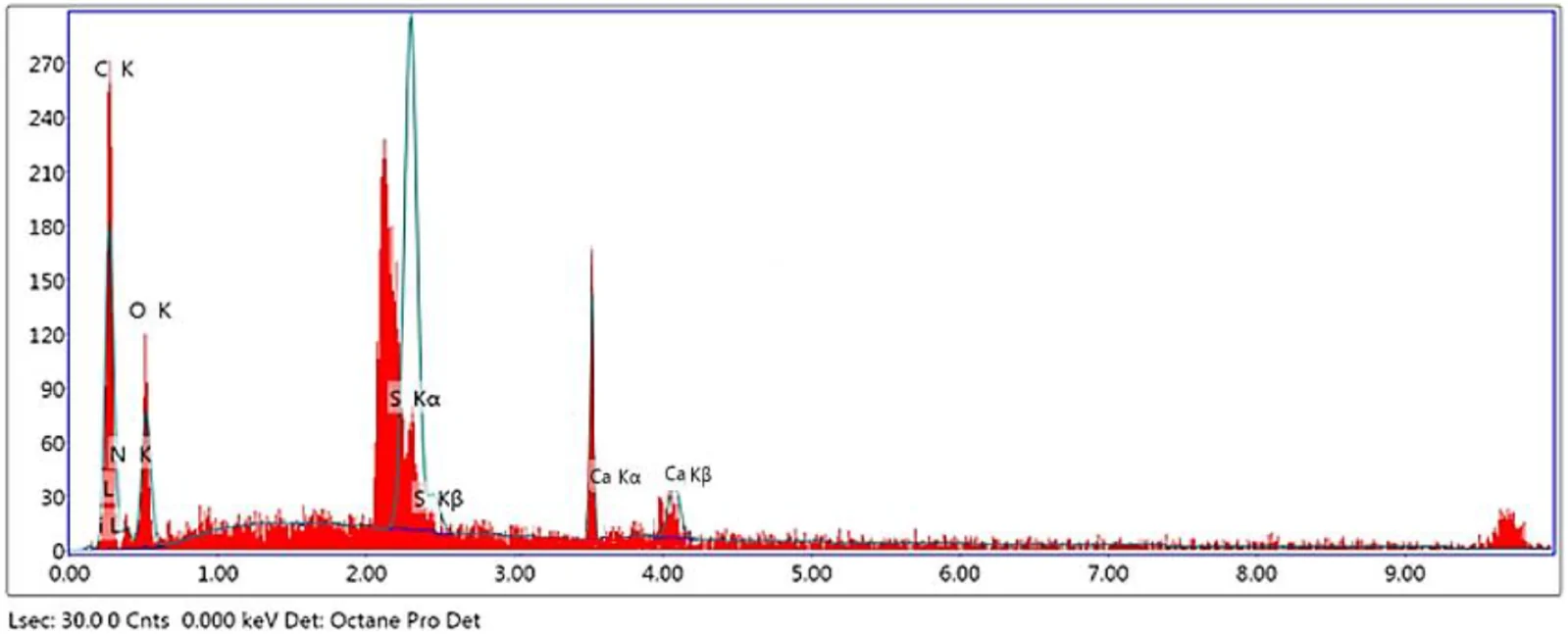
EDX spectra of the chitin-*g*-poly­(VESL/VIm)/CaCO_3_/glycerol nanocomposite hydrogel.

### 3.9 Rheological Properties of the Optimized Chitin-*g*-Poly­(VESL/VIm)/CaCO_3_/Glycerol Nanocomposite Hydrogel

The rheological properties of the synthesized hydrogel have been studied through the evaluation of the swelling ratio, mechanical strength, and viscoelastic properties, i.e., elastic/storage modulus (G’) and viscous modulus/loss modulus (G”) of different hydrogel concentrations as a function of temperature at different salinities of brine solutions. Furthermore, the loss factor of different hydrogel concentrations has been evaluated as a function of angular frequency at 160 °C and 350,000 ppm.

#### 3.9.1 Effect of Ionic Strength Response and Temperature

Unlike the majority of polyelectrolyte hydrogels that obey the Arrhenius law and demonstrate the thermothinning behavior of decreasing equilibrium swelling ratios with increasing temperatures either in pure water or saline solutions,^113^ the chitin-*g*-poly­(VESL/VIm)/CaCO_3_/glycerol nanocomposite hydrogel demonstrates a unique self-healing, thermoresponsive swelling behavior in a salt-free polymer solution as well as saline solutions up to 350,000 ppm with the increase of temperature. Figure 12a–b demonstrates the effect of salinity on hydrogel aqueous solution concentrations of 0.1 and 0.2 wt % at various temperatures ranging from 25 to 160 °C. As indicated in Figure 12a–b, chitin-*g*-poly­(VESL/VIm)/CaCO_3_/glycerol hydrogel nanocomposite solutions show a self-healing, thermoresponsive swelling behavior of the hydrogel system with the increment of the temperatures, where these properties are controlled by its amphoteric composition along with both hydrophilic and hydrophobic interactions. At temperatures below the UCST, an initial stabilization of the equilibrium swelling ratio was observed, which can be attributed to strong hydrogen bonding between polymeric chains along with electrostatic interactions between sulfonate anions and protonated imidazole groups, resulting in a compact polymeric network structure which hinders swelling of the polymeric hydrogel. With the increase in temperature reaching the UCST, these hydrogen bonds are weakened, providing mobility to the polymeric network while sustaining mechanical integrity, which facilitates water–hydrogel interactions, allowing water hydration of the polymeric network, and hence, a sharp increase in the swelling ratio can be observed, which characterizes the UCST-type hydrogels. Above the UCST and after reaching the maximum equilibrium swelling ratio, hydrophobic aggregation of lignin’s aromatic groups becomes more dominant, and partial chain hydrolysis of chitin provides hydrophilic groups, which leads to stabilized water uptake. It can be observed that the thermoresponsive swelling behavior became more pronounced as the aqueous solution salinity increased beyond 10,000 ppm. The UCST decreases with an increase in the salinity of the aqueous solution from 10,000 ppm to 350,000 ppm, where the UCST decreased from 60 to 20 °C as the salinity increased from 50,000 ppm to 350,000. The UCST values were systematically determined from the volumetric swelling curves using the transition onset criterion, defined graphically as the intersection point between the low-temperature baseline tangent and the tangent aligned with the steepest initial swelling slope. These results are superior compared to the chitin hydrogel, which demonstrates a significant decrease in the swelling ratio.^39^ This unique behavior of the synthesized hydrogel can be justified by the synergetic amphoteric design of the hydrogel, where sulfonated lignin contributes strong anionic charges and an aromatic hydrophobic domain, while VIm monomer provides cationic groups. With the increase in temperature, these associations are disrupted, allowing water intake. On the other hand, with increasing salinity of aqueous solutions from 10,000 ppm to 200,000 ppm, salt cations shield interchain ionic bonds, facilitating disruption of electrostatic attraction, hence decreasing the UCST from 60 to 20 °C, which results in improved swelling. With further increase of saline solution concentration up to 350,000 ppm, the salting-out effect reduces swelling values; however, the amphoteric balance prevents collapse, achieving high swelling. Furthermore, chitin’s hydroxyl and amide groups provide hydrophilicity and contribute to stabilizing swelling at elevated temperatures. On the other hand, glycerol contributes flexible cross-linking, and CaCO_3_ nanoparticles behave as an ionic bridge, which provides both thermoresponse and salt-response behavior along with the mechanical strength of the synthesized hydrogel.

**Figure 12:**
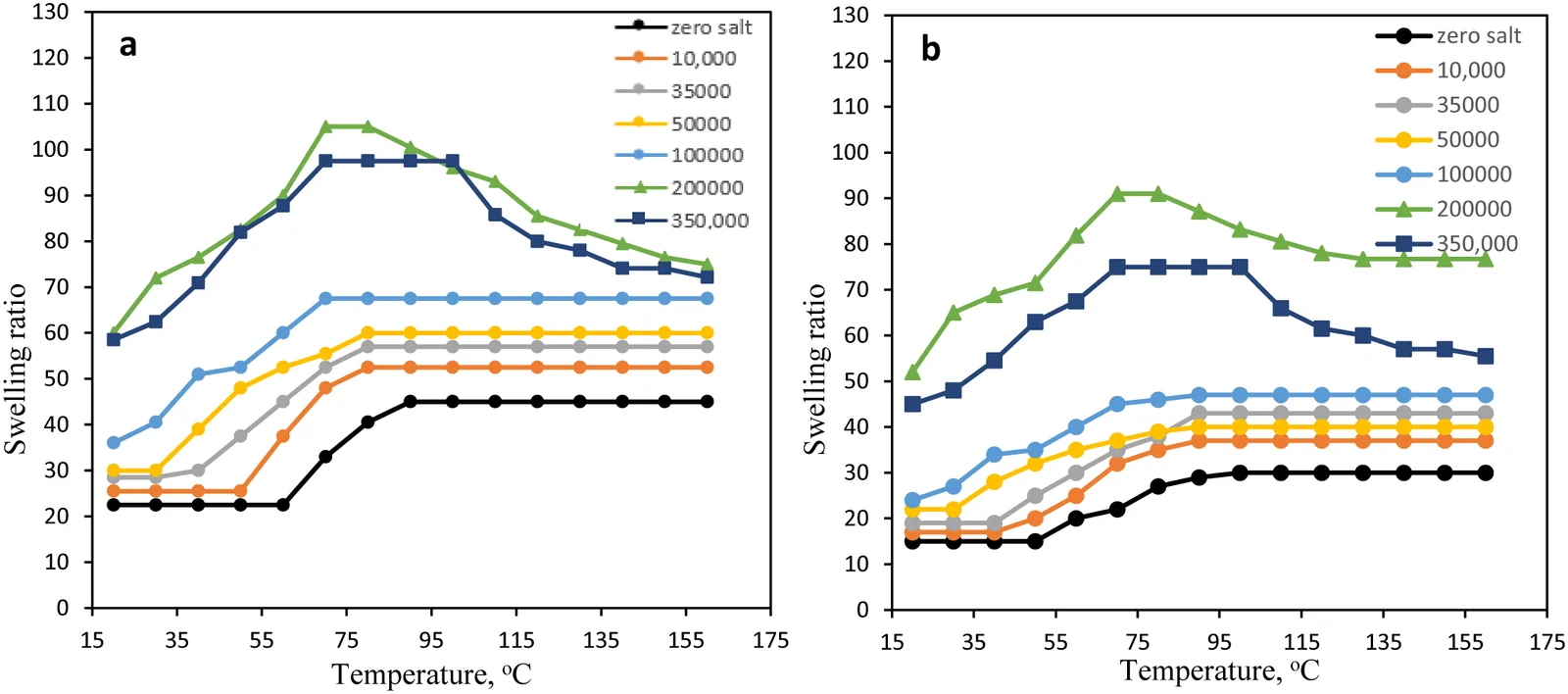
Swelling ratio versus temperature for chitin/poly­(VESL/VIm)/CaCO_3_/glycerol nanocomposite hydrogel solution in different saline solution concentrations: (a) Cp = 0.1 wt % and (b) Cp = 0.2 wt %.

Figure 13a indicates the equilibrium swelling ratio of different chitin-*g*-poly­(VESL/VIm)/CaCO_3_/glycerol hydrogel concentrations (0.08, 0.1, 0.2, 0.3, and 0.5 wt %) at 160 °C and 350,000 ppm synthetic water. As demonstrated in Figure 13a, there is a high dependency of the equilibrium swelling ratio on the hydrogel concentrations. Furthermore, a clear thermoresponsive behavior is notable along with all the investigated hydrogel concentrations, even at ultralow hydrogel concentrations of 0.08 wt %. The hydrogel concentration of 0.08–0.10 wt % demonstrates the highest equilibrium swelling ratio. The behavior can be attributed to high chain mobility at low hydrogel concentration owing to the disturbance of hydrogen bonds and imidazolium–sulfonate pairs with increasing temperatures, which produces an increase in the swelling at relatively low UCST values of ≈20 °C. With further increase in temperature, a maximum equilibrium swelling ratio is reached, followed by a plateau owing to higher free volume and reduced content of hydrophobic lignin that restricts the hydrophobic aggregations induced by increasing temperatures. It can also be observed that the maximum equilibrium swelling ratio systematically decreases as the hydrogel concentration increases from 0.08 to 0.5 wt %. The behavior can be attributed to the increased chain density and cross-link volume per hydrogel with the increase of hydrogel concentration, which requires a higher temperature to disrupt the hydrogen bonding. Therefore, a denser polymer decreases the free volume per polymer concentration, resulting in a decrease in the maximum swelling equilibrium. It is also notable that the swelling values at hydrogel concentrations higher than 0.08 wt % decrease at temperatures higher than 90 °C. This can be attributed to the dominating salting-out effect resulting from excessive hydrophobic aggregation of lignin aromatics at elevated temperature.

**Figure 13:**
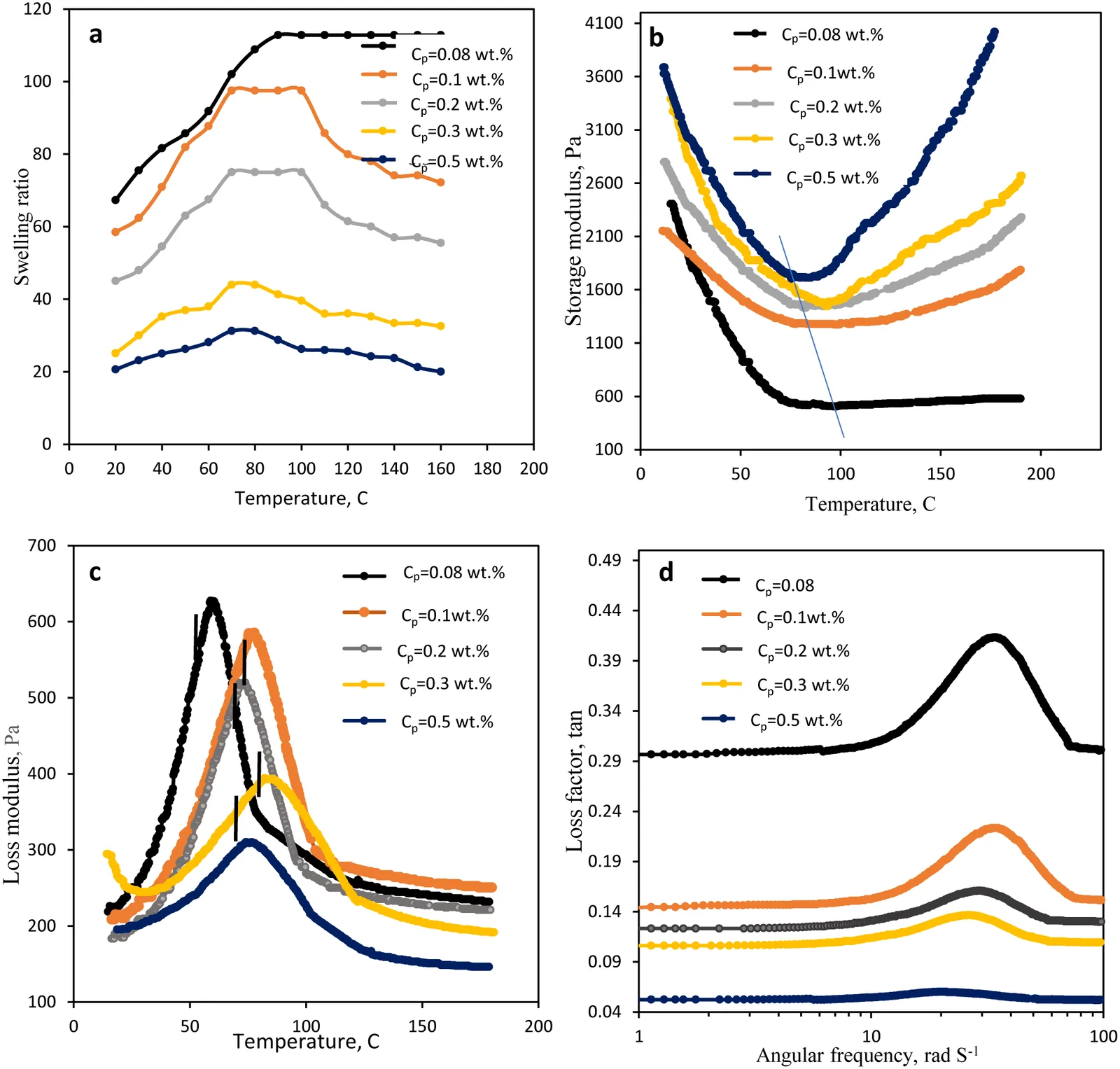
(a) Swelling ratio as a function of temperature for different polymer nanocomposite concentrations (C_p_) at 350,000 ppm and 160 °C; (b) storage modulus as a function of temperature for different polymer nanocomposite concentrations (C_p_) at 350,000 ppm and 160 °C; (c) loss modulus as a function of temperature for different polymer nanocomposite concentrations (C_p_) at 350,000 ppm and 160 °C; (d) loss factor for different polymer nanocomposite concentrations (C_p_) at 350,000 ppm and 160 °C.

Figure 13 (b and c) indicates the storage and loss modulus of different hydrogel concentrations for hydrogel concentrations ranging from 0.08 to 0.5 wt % evaluated at 350,000 ppm and 160 °C. As demonstrated in Figure 13b, the storage values increase with increasing hydrogel concentration. At low temperatures, the synthesized hydrogel demonstrates high storage modulus values, which initially decrease with increasing temperature, where the thermal energy disturbs the hydrogen bonding and weakens the intermolecular interaction of imidazolium groups and lignin sulfonates and decreases the effective cross-link density and storage modulus. With further increase in temperature, a plateau of the storage modulus is observed, demonstrating the balanced effect of hydrophobic aggregation of lignin aromatic groups and salting-out under extreme ionic strength and chain thermal hydrolysis. This G′ plateau is followed by an increase in G′ with temperature. The behavior is attributed to the pronounced hydrophobic aggregation of lignin owing to proximate chains; hence, new physical junctions form that restore some elastic modulus. Figure 13c indicates an initial increase in the viscous modulus with the increase of temperature, forming a pronounced viscous modulus peak. Higher values were observed for higher hydrogel concentrations, where chain rearrangements increase viscous dissipation. Further, the viscous modulus curves decline, demonstrating decreased viscous rearrangement owing to the formation of hydrophobic aggregates. As shown in Figure 13 (b and c), higher G′ values than the G″ of the investigated hydrogel concentrations demonstrate the presence of the synthesized hydrogel in a gel form as the temperature varies from 25 to 160 °C. Furthermore, the mechanical performance of the synthesized hydrogel improved with increasing concentration; however, this increases the chance of excessive aggregation at high temperatures and extreme salinity.

Figure 13d demonstrates the loss factor of different hydrogel concentrations evaluated at 160 °C and 350,000 ppm and indicates the loss factor values as a function of angular velocity for different hydrogel concentrations of 0.08, 0.1, 0.2, 0.3, and 0.5 wt %. As shown in Figure 13d, the loss factor values demonstrate an initial increase with increasing angular frequency and continue increasing until reaching a maximum, followed by a decline with any further increase in frequency or decrease. Furthermore, it can be observed that higher hydrogel concentrations show a lower, broader peak at a lower frequency range and temperature. The behavior can be attributed to the presence of more hydrophobic segments of lignin at higher hydrogel concentrations that tend to aggregate at lower temperatures; hence, it shows a higher storage modulus at lower angular velocity, and loss factor values decrease across frequencies. The loss ranged from 0.152 to 0.293, which indicates that the elastic criteria of the synthesized hydrogel are higher than the viscous criteria, which demonstrates higher viscoelastic properties compared to chitin, as reported.^114^ The behavior can be attributed to reduced flexibility of the polymeric chain due to the presence of the glycerol cross-linker and CaCO_3_ nanoparticles^115^ and hydrophobic segments of lignin that result in a decrease in the damping factor.^116^

### 3.10 Core-Flooding Experiments

Three fractured carbonate cores with approximately the same permeability were used to evaluate the plugging performance of the synthesized chitin-*g*-poly­(VESL/VIm)/CaCO_3_/glycerol hydrogel nanocomposite at reservoir conditions at 350,000 ppm and 160 °C. To ensure experimental repeatability and evaluate data uncertainty, each core-flooding condition was repeated three times under identical experimental conditions. Further, the standard deviation (SD) and the relative standard deviation (RSD%) were calculated for each core-flooding experiment. The evaluation was based on the pressure differential profile attained from flooding experiments and the corresponding RRF values. The synthesized self-healing nanocomposite hydrogel demonstrated an RRF of 300.5 ± 5.7 (RSD = 2.1%) at 0.08 wt % and 191.1 ± 4.8 (RSD = 2.6%) at 0.1 wt %, whereas the native chitin hydrogel demonstrated a significantly lower RRF of 5.8 ± 0.3 (RSD = 5.2%). The synthesized self-healing nanocomposite demonstrated a pioneering plugging efficiency of 99.7 ± 0.4% and 99.5 ± 0.5% for the 0.08 and 0.1 wt % concentrations, respectively. Figure 14 demonstrates the flooding pressure drop as a function of injected pore volume for brine, chitin, and self-healing hydrogel concentrations of 0.08 and 0.1 wt %. Notably, the pressure differential was almost constant during brine injection. When the self-healing nanocomposite was injected, the pressure differential initially increased with the injected pore volume, then dropped and stabilized. As indicated in Figure 14, the higher the nanocomposite hydrogel concentration, the lower the injected pore volume needed for the stabilization of the differential pressure, demonstrating improved plugging performance and delayed water breakthrough at lower concentrations of the injected nanocomposite hydrogel. The native chitin hydrogel was extruded from the fractured core, evidenced by the differential pressure stabilization after approximately 0.3 PV, while the self-healing nanocomposite hydrogel demonstrated a high resistance to brine, with differential pressure stabilization occurring after 1.5 PV and 0.8 PV for concentrations of 0.08 and 0.1 wt %, respectively. The initial pressure increase can be attributed to the absence of open pathways owing to hydrogel swelling. The following abrupt pressure drop is assigned to the formation of new permeable paths. The behavior is aligned with the work reported by He et al.^117^ At higher nanocomposite concentrations, the hydrogel shows a reduction in swelling owing to increased cross-link density, resulting in a slightly lower pressure differential; nevertheless, enhanced mechanical stability is achieved.. This justifies the maximum RRF and the highest plugging efficiency at a nanocomposite concentration of 0.08 wt %. Meanwhile, the native chitin hydrogel displayed the lowest RRF and differential pressure (5.8, 48 psi), owing to its weak mechanical integrity and poor resistance to swelling at HTHS reservoir conditions, as evidenced in previous literature by Shibu et al.^114^ As summarized in Table 9, a direct comparison with recent water-control hydrogels highlights the distinct advantages of the novel sustainable nanocomposite hydrogel with respect to composition, thermal stability limit, salinity resistance, and plugging efficiency. While recent work has largely depended on synthetic polyacrylamide or poly­(vinyl alcohol) (PVA) backbones employing 2D nanofillers or dual cross-linked networks, recent literature has formulated liquid gelants using amino acid or organic cross-linking pairs. Although these synthetic architectures can maintain stable performance thresholds of up to 80–150 °C and up to 225,000 ppm salinity conditions, they are still highly vulnerable to extremely severe ionic charge shielding. This vulnerability arises from the flexibility of linear synthetic chains, which readily coil and precipitate when extremely aggressive monovalent and divalent ions screen their electrostatic repulsive forces, ultimately causing network collapse and water syneresis.

**Figure 14:**
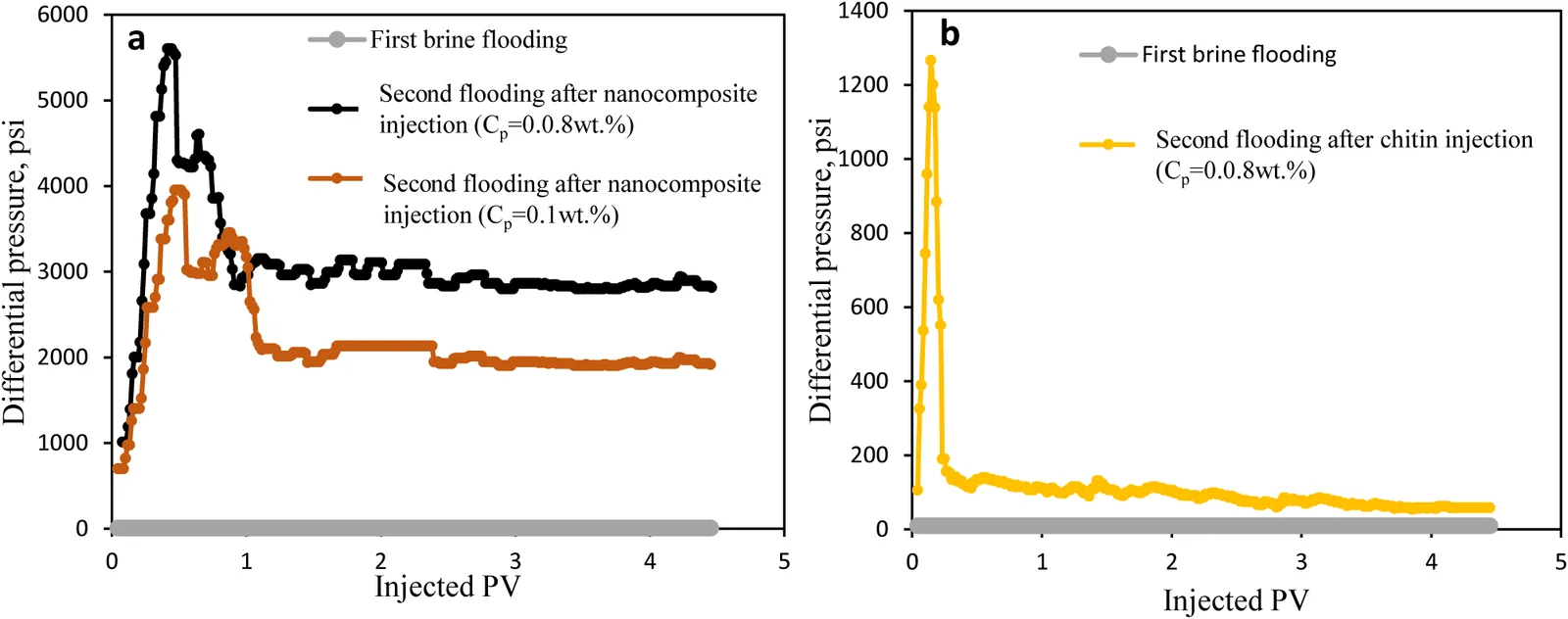
Differential pressure as a function of injected pore volume: (a) self-healing nanocomposite concentrations of C_p_ = 0.08 wt % and C_p_ = 0.1 wt % and (b) chitin flooding experiment of C_p_ = 0.08 wt %.

**Table 9:** Comparison between the Thermal and Chemical Stability Limits, Gelation Time, Storage Modulus, and Plugging Performance Reported by Previous Studies and the Current Study

Published work	Polymer composition	Salinity tolerance, ppm	Thermal resistance, °C	Gelation time, hr	Long-term stability, weeks	Storage modulus, Pa	Plugging efficiency, %
^73^	Graphene/sodium silicate polyacrylamide	200,000	80	30	2	58,100	97–99.5
 ^62^	Nanosilica/starch-graft-polyacrylamide	225,000	90	3.5	1	900	N/A
^118^	Nanosilica/aluminum-cross-linked polyacrylamide	50,000	100	0.83 to 1.33	0.14	2340	N/A
^119^	l-Lysine/polyacrylamide (PAM/lysine)	200,000	130	72	28.57	400	N/A
^120^	Thiourea/nano-(SiO_2_)/AMPS-PAM/PEI	160,000	110	60 to 84	8	N/A	>95% water plugging (selective; <35% gas impairment)
^121^	Self-healing 2D-nanofiller/polyacrylamide (PAM/GO/CG/BN)	20,000	150	48	1.1	2970	N/A
^122^	Tannic acid/DA-CNF/AFBN/PAM/PVA	200,000	130	N/A	30	N/A	N/A
**Current study**	**chitin-*g*-poly (VESL/VIm)/CaCO_3_/glycerol nanocomposite hydrogel**	**350,000**	**160**	**0.05**	**12**	**1700**	**99.7**

By contrast, the formulation of the newly synthesized self-healing nanocomposite hydrogel achieves unique stability and plugging performance at 160 °C and 350,000 ppm TDS with absolute zero syneresis by completely reimagining the macromolecular matrix. Through grafting the green aromatic VESL and VIm network onto a crystalline chitin core, reinforced with in situ CaCO_3_ nanoparticles and green recycled glycerol, this tailored amphoteric composition induces a salt-responsive antipolyelectrolyte effect. Rather than coiling, the chains expand and stiffen in saltwater. In addition, the rigid polysaccharide backbone of chitin and the aromatic rings of VESL supply strong steric hindrance against thermal degradation, while the embedded CaCO_3_ nanoparticles along with glycerol serve as rigid physical anchors that distribute mechanical stress under high shear. This distinctive formulation enables the low-viscosity polymer solution to be seamlessly injected under surface conditions into extreme HTHS reservoir conditions, forming a robust downhole plugging at extreme HTHS conditions. It sustains a 1,700 Pa storage modulus and a 99.7% ± 0.4% plugging efficiency with zero syneresis at an unprecedented upper limit of 160 °C and 350,000 ppm TDS, using an ultralow concentration of only 0.08 wt %.

## 4 Conclusions

This study demonstrates, for the first time, a sustainable, self-healing, and thermoresponsive chitin-based nanocomposite hydrogel synthesized through a fully green, near-zero-waste route for water shutoff applications in ultradeep, extreme HTHS reservoirs of 160 °C and 350,000 ppm salinity. Shrimp shells were valorized into chitin and CaCO_3_ nanoparticles *via* a CO_2_-switchable NADES-supported enzymatic extraction, an approach not previously reported in the literature, followed by their copolymerization with a VESL macromonomer derived from lignin and upcycled glycerol as a green cross-linker. This integrated strategy provides an innovative, sustainable, near-zero-waste synthesis route for the synthesis of robust bulk gels in extreme HTHS conditions. RSM has been utilized to tailor the best formulation of the chitin-*g*-poly­(VESL/VIm)/CaCO_3_/glycerol nanocomposite hydrogel for further utilization in water shutoff application at 160 °C and 350,000 ppm salinity. The developed quadratic models have been utilized to evaluate the effect of the process parameters (chitin concentration, VESL:VIm ratio, CaCO_3_ loading, and glycerol content) on the responses (swelling capacity and viscoelastic properties) under these conditions. The results demonstrated that the best formulation for the chitin-*g*-poly­(VESL/VIm)/CaCO_3_/glycerol nanocomposite hydrogel was 35.8 wt.% chitin concentration, 45:1 VESL/VIm molar ratio, 3.5 wt.% CaCO_3_ loading, and 9.1 wt.% glycerol concentration, where the highest swelling ratio and storage modulus were 78.2 and 1700 Pa under optimum conditions. The optimized nanocomposite hydrogel has demonstrated a superior swelling ratio and storage modulus of 80.2 ± 1.5 and 1700.4 ± 12 Pa using ultralow concentrations of 0.08 wt %. Core-flooding experiments confirmed the ability of the nanocomposite hydrogel to reduce water permeability in carbonate cores, achieving an RRF value of 300.5 ± 5.7 and high plugging efficiency of 99.7% ± 0.4% using an ultralow hydrogel concentration of 0.08 wt.% at 160 °C and 350,000 ppm. Therefore, this study presents an innovative strategy for the development of a green nanocomposite hydrogel that integrates self-healing and thermoresponsive functionalities with strong sustainability credentials. The resulting material demonstrates significant potential as a water shutoff agent for ultradeep reservoirs operating under extreme HTHS conditions. Importantly, the near-zero-waste valorization pathways employed in this work introduce a viable and scalable route for converting seafood waste, agro-industrial lignin residues, and biodiesel byproducts into high-value polymeric materials, thereby contributing to the advancement of a circular bioeconomy.
